# Supplementary material for: A pair of effectors encoded on a conditionally dispensable chromosome of Fusarium oxysporum suppress host-specific immunity
Source: Commun Biol. 2021 Jun 9;4:707. doi: 10.1038/s42003-021-02245-4 (PMC8190069; doi:10.1038/s42003-021-02245-4)
Supplement: Supplementary file 2 — Supplementary Information [file 42003_2021_2245_MOESM2_ESM.pdf]

Supplementary Information

A pair of effectors encoded on a conditionally dispensable chromosome of *Fusarium oxysporum* suppress host-specific immunity

Yu Ayukawa, Shuta Asai, Pamela Gan, Ayako Tsushima, Yasunori Ichihashi, Arisa Shibata, Ken Komatsu, Petra M. Houterman, Martijn Rep, Ken Shirasu & Tsutomu Arie

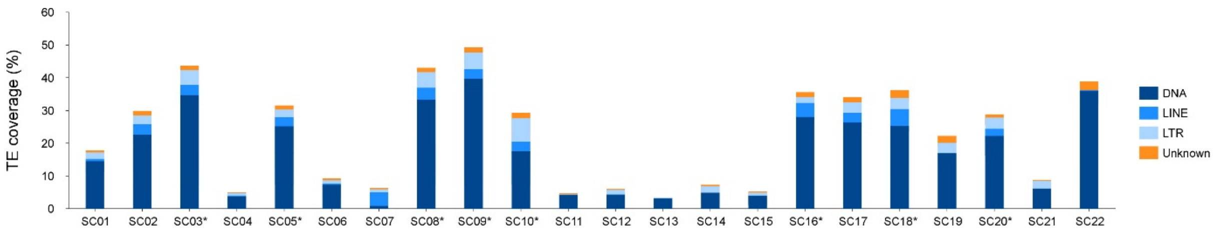

Supplementary Figure 1 | Proportions of sequences associated with TEs.

Proportions of sequences of respective SCs associated with DNA transposons, retrotransposons (LTR and LINE) and unknown repeats. Distribution of TEs is shown in Figure 1a. Asterisks indicate dispensable genomic regions in *Focn*Cong:1-1.

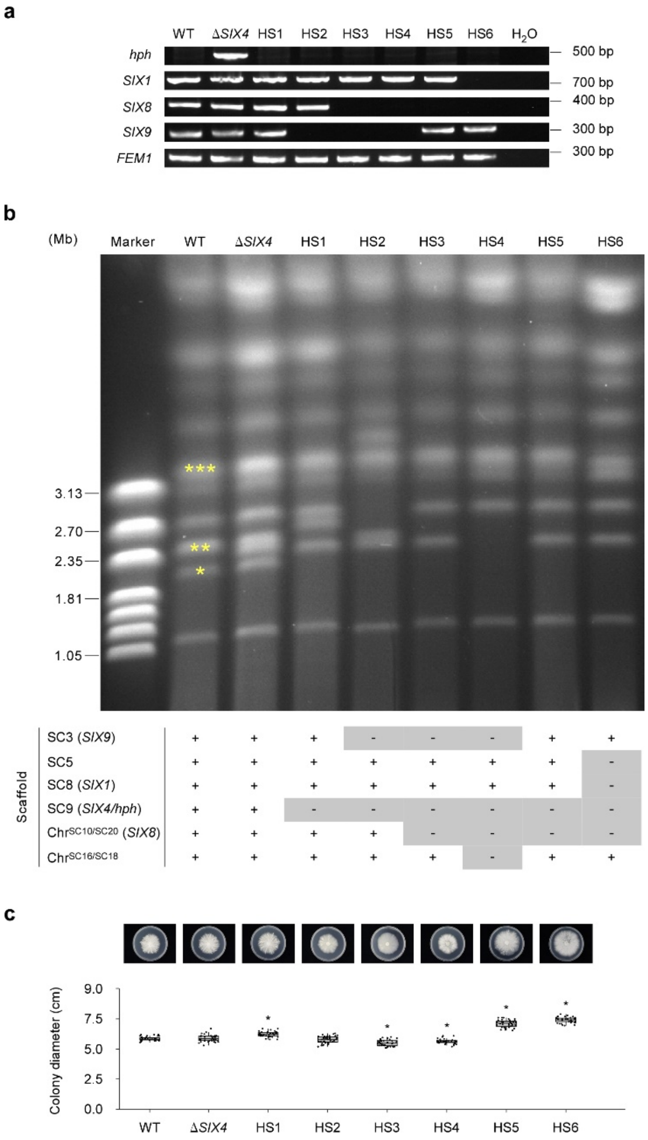

Supplementary Figure 2 | Effects of loss of dispensable chromosomes on radial growth in *Focn*Cong:1-1.

a, Detection patterns of *SIX* genes in hygromycin B-sensitive *Focn*Cong:1-1 mutants (HS1 to HS6). Hygromycin B resistance gene (*hph*), *SIX1*, *SIX8* and *SIX9* genes were detected by PCR. *FEM1* amplification was used as a control. DNAs of *Focn*Cong:1-1 WT, Δ*SIX4* and HSs were used as a template. **b,** Electrophoretic karyotype of *Focn*Cong:1-1 WT, Δ*SIX4* and HSs. Asterisks indicate chromosomes on which *SIX* genes are located as follows: **SIX4*, ***SIX8*, ****SIX1*. SC loss patterns estimated by genome sequencing described in Figure 2a are shown as a table. + and – represent maintained- and lost-SCs, respectively. *SIX*s located on SCs are

shown in parentheses. **c,** Colony formation of *Focn*Cong:1-1 WT, Δ*SIX4* and HSs on potato dextrose agar. Results of six independent experiments were combined and a total of 35 or 36 biological replicates are plotted. Boxplots indicate median value, estimated 25th and 75th percentiles, and whiskers represent 1.5 times the interquartile range. Asterisks represent significant difference from Δ*SIX4* (**p* < 0.0001, Welch's t-test). Representative images of colonies after 8 days of incubation are shown above each boxplot.

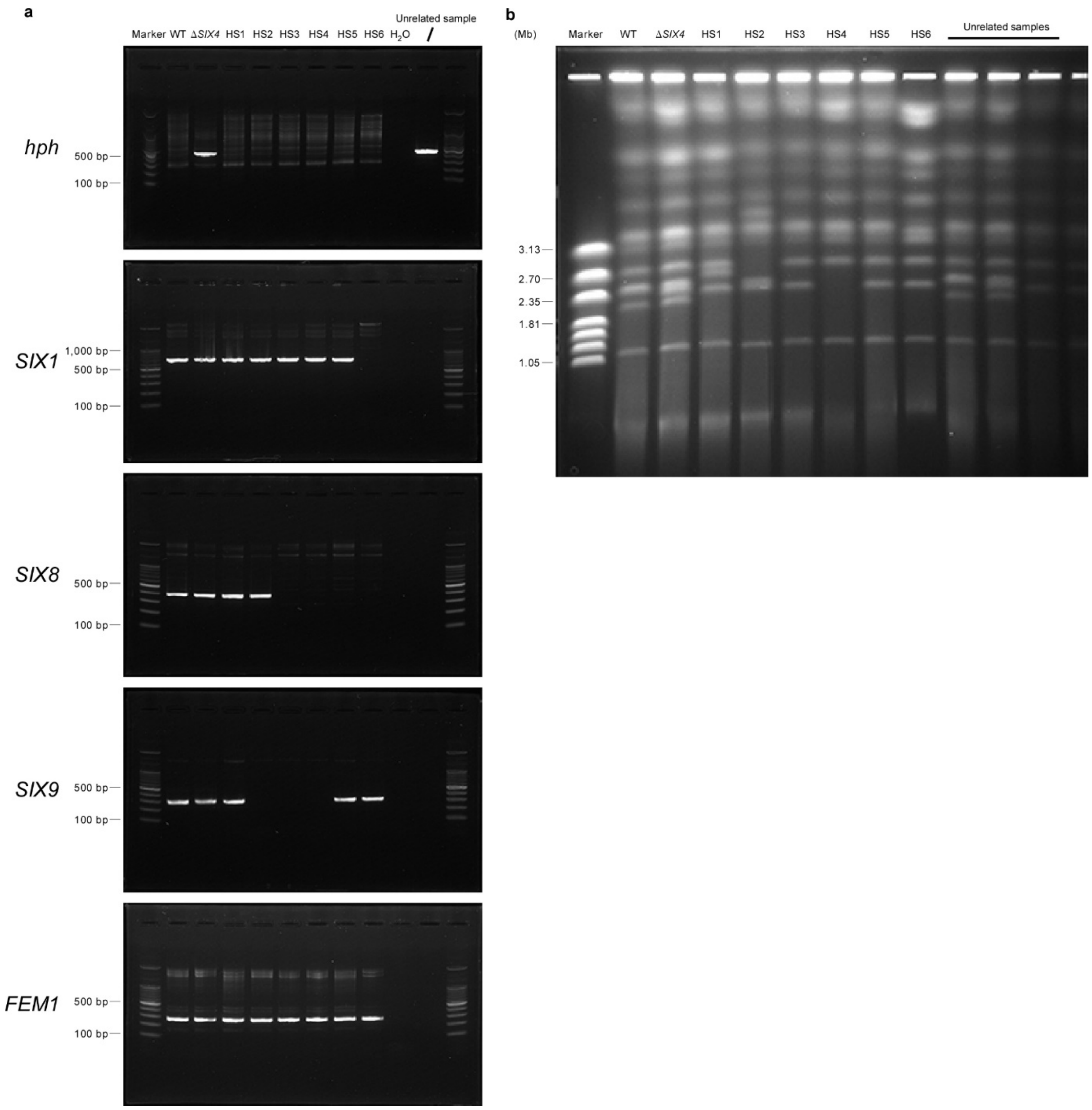

Supplementary Figure 3 | Uncropped gels from Supplementary Figure 2.

a, Uncropped gels from Supplementary Figure 2a. **b,** An uncropped gel from Supplementary Figure 2b.

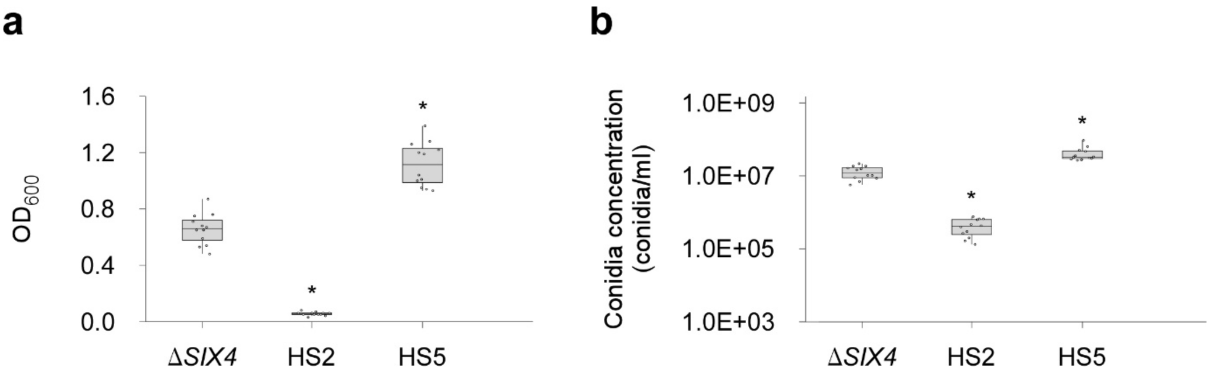

Supplementary Figure 4 | Comparison of measurement methods for conidial formation. Conidial formation in *Focn*Cong:1-1Δ*SIX4*, HS2 and HS5. Conidial suspensions were prepared from six colonies after 17 days of incubation on potato dextrose agar. The amount of conidia was quantified by measuring OD_{600} value of conidial suspensions (**a**) and by counting using haemocytometer (**b**). Results of two independent experiments were combined and a total of twelve biological replicates per isolate are plotted. Boxplots indicate median value, estimated 25th and 75th percentiles, and whiskers represent 1.5 times the interquartile range. Asterisks represent significant differences from *Focn*Cong:1-1 Δ*SIX4* (*$p < 0.001$, Welch's t-test).

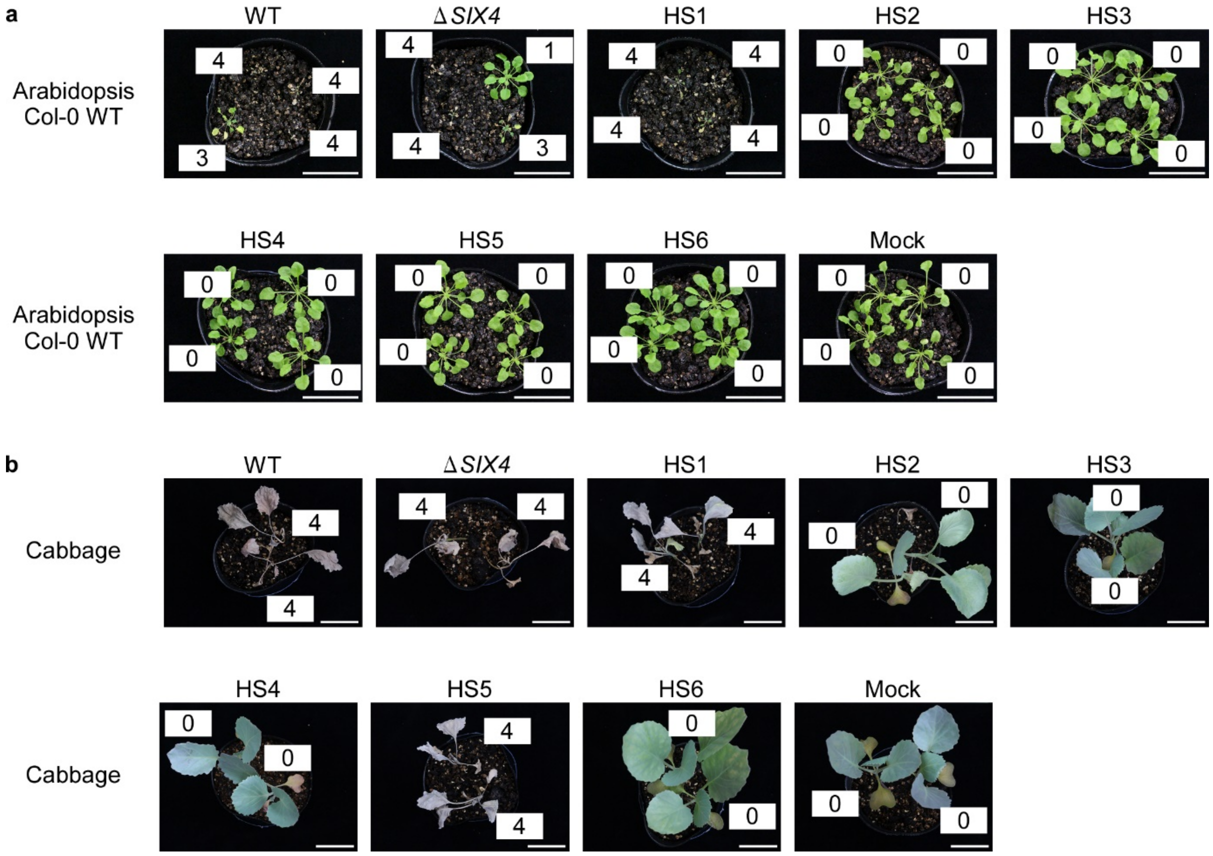

Supplementary Figure 5 | Representative images of infected plants from Figure 2d. Representative images of infected Arabidopsis Col-0 WT (**a**) and cabbage (**b**) described in Figure 2d with disease index scores. Scale bars indicate 3 cm.

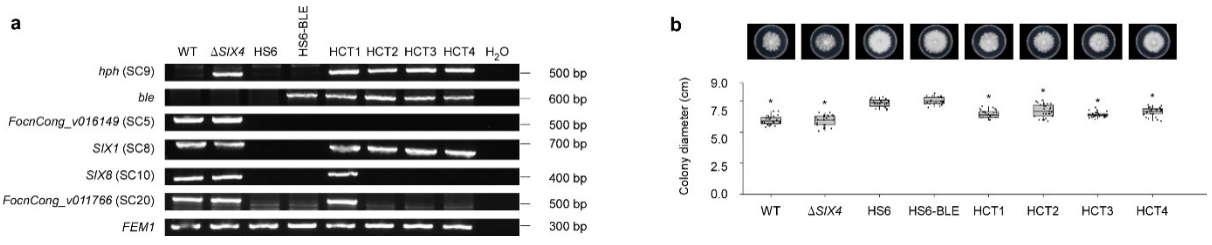

Supplementary Figure 6 | Effects of chromosome transfer on radial growth in *Focn*Cong:1-1 HS6.

a, Detection patterns of genes located on particular scaffolds (SCs) in horizontally chromosome-transferred recipient colonies (HCT1 to HCT4). Hygromycin B resistance gene (*hph*), zeocin resistance gene (*ble*), *FocnCong_v016149*, *SIX1*, *SIX8* and *FocnCong_v011766* were detected by PCR. *hph*, *FocnCong_v016149*, *SIX1*, *SIX8* and *FocnCong_v011766* are located on SC9, SC5, SC8, SC10 and SC20, respectively. *FEM1* was amplified as a control. DNAs of *Focn*Cong:1-1 WT, Δ*SIX4,* HS6, HS6-BLE and HCTs were used as a template. **b,** Colony formation of *Focn*Cong:1-1 WT, Δ*SIX4,* HS6, HS6-BLE and HCTs on potato dextrose agar. Results of four independent experiments were combined and a total of 25 biological replicates are plotted. Boxplots indicate median value, estimated 25th and 75th percentiles, and whiskers represent 1.5 times the interquartile range. Asterisks represent significant difference from HS6-BLE (*$p < 0.0001$, Welch's t-test). Representative images of colonies after 8 days of incubation are shown above each boxplot.

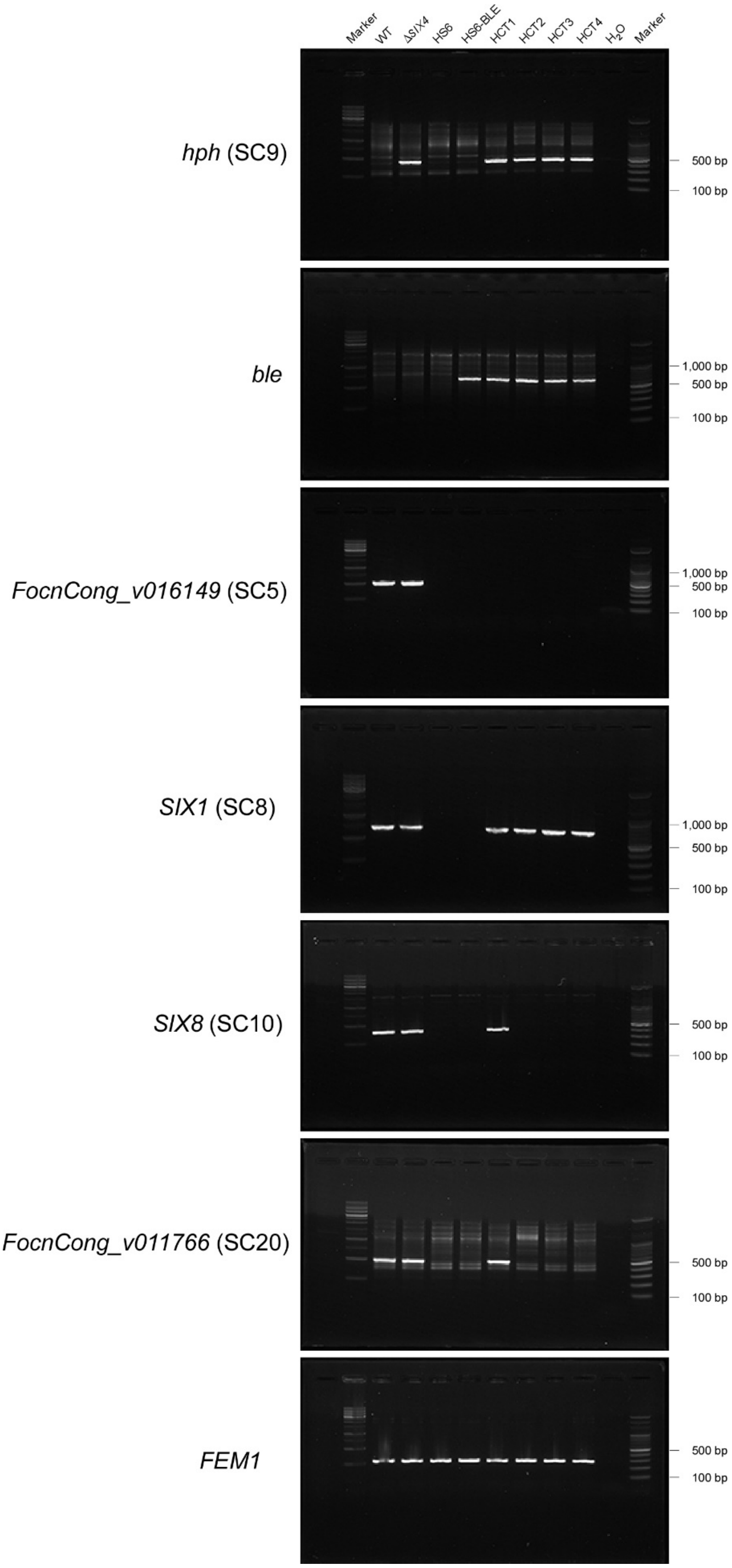

Supplementary Figure 7 | Uncropped gels from Supplementary Figure 6a.

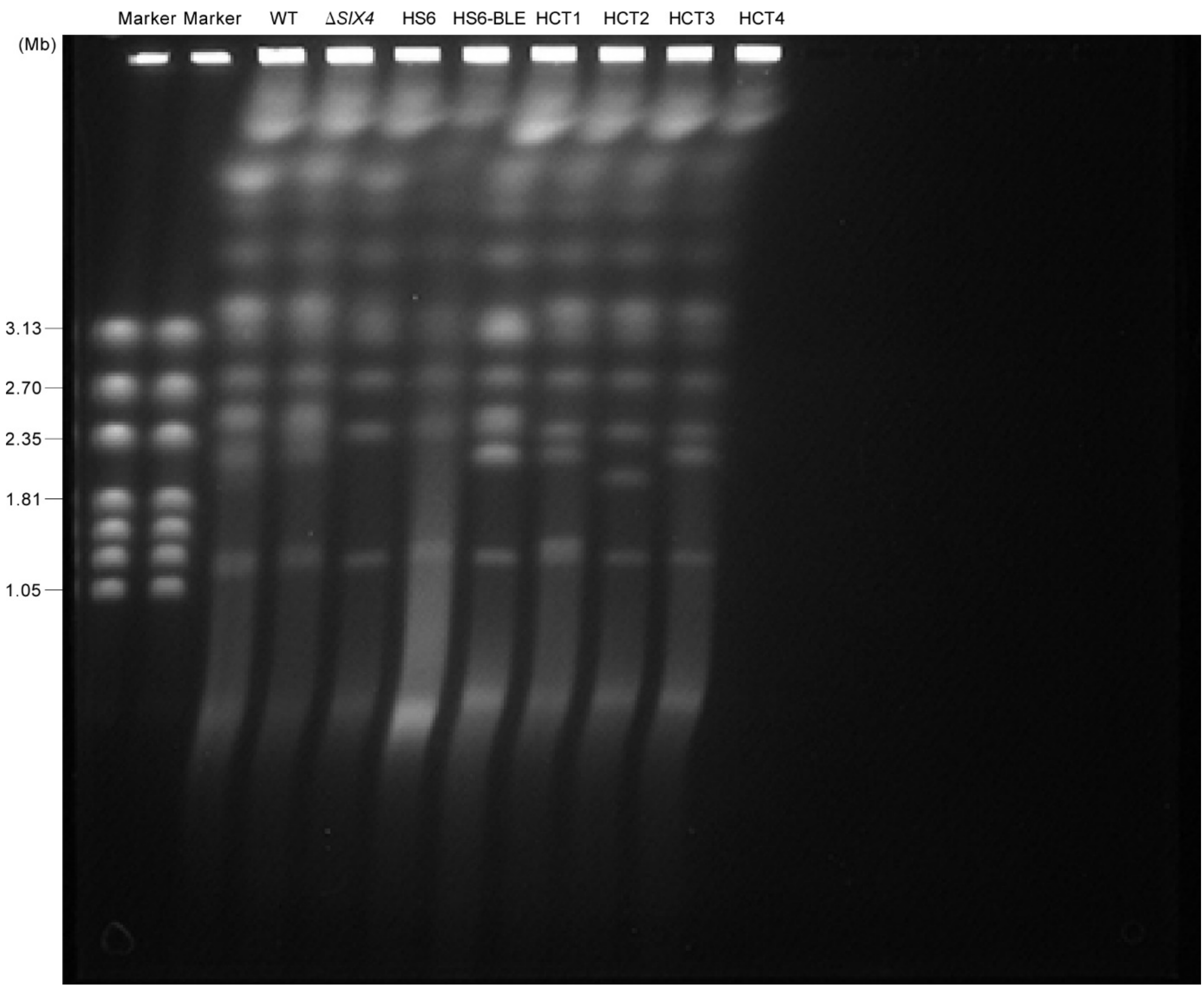

Supplementary Figure 8 | An uncropped gel from Figure 3a.

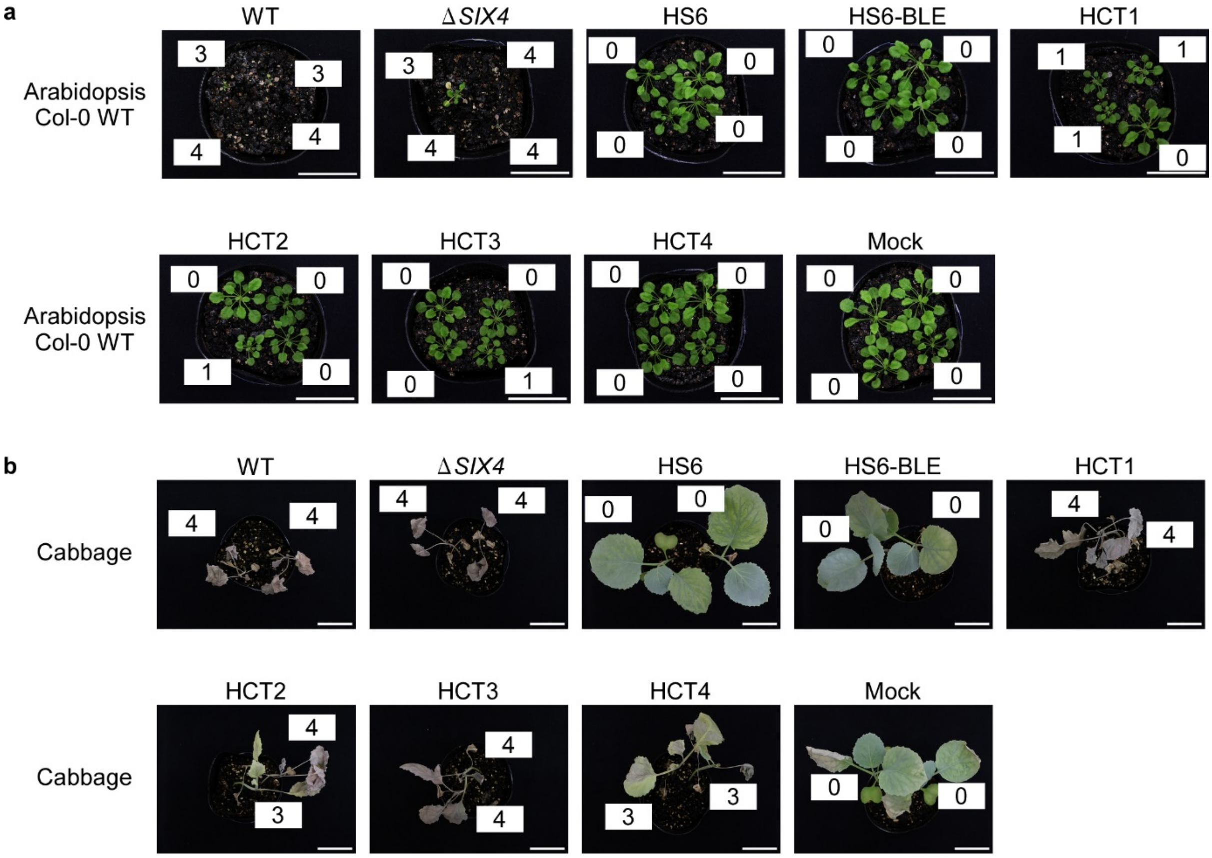

Supplementary Figure 9 | Representative images of infected plants from Figure 3c. Representative images of infected Arabidopsis Col-0 WT (**a**) and cabbage (**b**) described in Figure 3c with disease index scores. Scale bars indicate 3 cm.

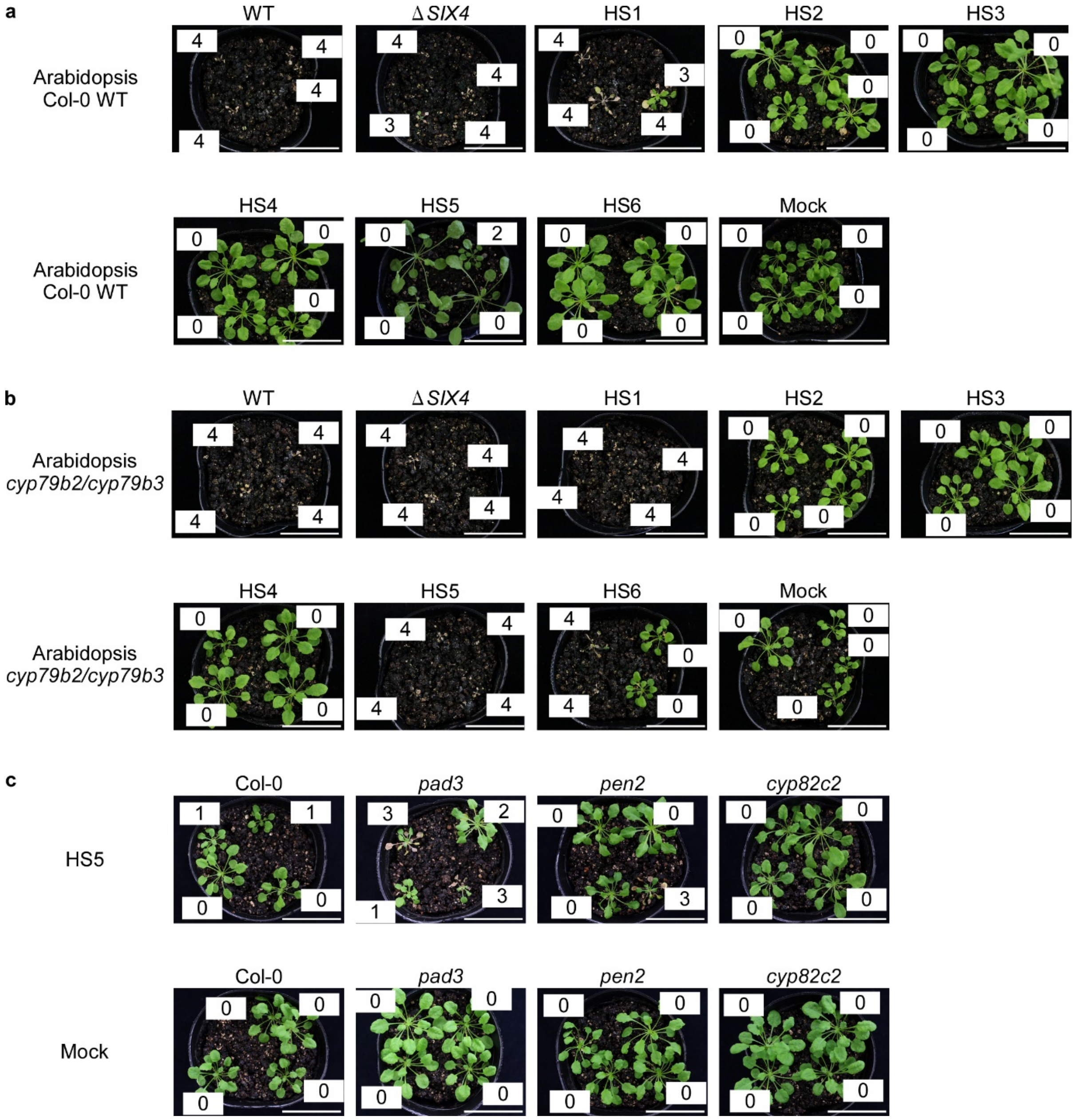

Supplementary Figure 10 | Representative images of infected plants from Figure 4a, d.

Representative images of infected Arabidopsis Col-0 WT (**a**) and *cyp79b2/cyp79b3* (**b**) described in Figure 4a with disease index scores. **c,** Representative images of infected Arabidopsis Col-0 WT, *pad3*, *pen2* and *cyp82c2* described in Figure 4d with disease index scores. Scale bars indicate 3 cm.

a

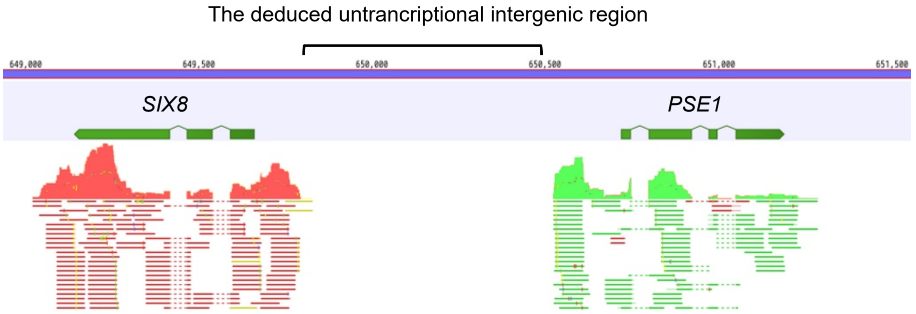

b

>*Focn*Cong:1-1_SC10:648500-652000

```
TAGTCGCGCCTCAGGATGAACGCATAAGGAGAGCGACTCGAGAAATTCATAAGGCGATTGTTGTATCCCTGTAGTTGGGGCAATAGGTGACGAT
TCCATGGTGTTAGCCCTTTCTGTGCTGTTATATACTGTTGCTGCTTCTATATTGTATTCTTTTATTATACGATATCGGGAGGCTGGAGCAGAAG
GCGCAACGGTAGAATATGGTAGGAATGGAATAAAAGTGGGAAGTCAGAAGCACGAGCGCGCACGGATAAAAAGGAGGTAAACAACAGGTTGTTT
TTATTAAACCTTCTTACCATGTCAATGCGCACGGACAAAAAGTATAGAAGCTACAGGCGATTTTACGGCAAAACGCCGGGGAAATTTGTAGTCA
CCAGGGTTGGCACGGACGAAAATCAAATAATGCGGCGCGATTTGGATGAACATTGGTACAGTAAGTAGGTTTCCATTCCACAAATTCCACAATA
TGGATTCATTTCCATAATGGATCCATTTCCACCCATTCCACATCGAAGTTATTAGTCAGCATCCATATCCACGCCATATTCACAAATGTGTCGT
GGATTTGAACCCTGGCCGCACCTGCTTGCTCCTTATCCCTATCGGGCCTAATCCACACAACACACAACACTAGCTACTAGAAATTGTTTATAAA
CTGGACAGTCGATGCACCTAAAGTAGCGATGTTCCTCACCGCACCCGGCACGATTTGGACTACCTCGTGTACCGCTTGTGAGAGCGCTCCCCCA
CTCAAAATAACAAGCACGTTCGATGTCCCTCCGCCCCTAGTTCTGTCCATCCTCAACGTAAGGGTGCGCGGGTCATTACGAAATGCAGTGAGAT
AGCATCCTGCATATGCCTGAAACGCCACACCGGCACCTGTTATGCAGGCGAGTAAAATGCCACTGGTGTCGCTGTGATTTACATTAGAAGCAAC
GTCAAATCTATACTAAATTACCTACCGTTCGACAAGGGCTCTCTCGTCATGAGAATGAGGTTGATGAACAGTTTCTTCAATAATGGCGGCTTGG
TCGAGGCTTTATATATTAATGAGCGTGACGGACGTATTCATGAGAATCAGACATACCTCTTATCAATATGCGTAGCAGCCACAGAGACGGCTAA
GGGGAAGAGTAAAAGAACGCGTAGGGGTTGCATAGCTGCCGTATATAATAGAGAGGGTATAAGTGAGGACGTAAAGACTTATTTAGGAAGCCGA
ACTAAGGGGAAAGTAAACCCGGACTCTTGATGCGGTACGAAAGGATGTGCGGGAACGACGAAATAGAGGGAGAGTCTAGCATTATATCTTTAGT
CTTATATGGCGCACTTCGCTGCTTAAATCTCTCCTAAAAGCTGTTCTTTTGATACAGGTAGAGTATAAAGTATAATTACCCTGTAACATAATTT
AAAAAGCTCCATTCTTTGTTATTGCAGGGTATATTTTAAAGTTCATGTAAAGCAAAATTTAATTACACTAGGGTATCGAGCGCCCGCAAACACT
TTCCTGCTCGGTATCGCTCTTAAAAGTTACCTTCTCGGTCTTAATATTACATAAGACCGGCAGATAAATCAAATCGGCAGATGAACACGACCGA
CAGATATGTATGTAAGAAGGATCTTATTCGGCTAGACCGGCAGATAAATCACATCGGCAGATGAACACGACCGACAGATATGTATGTAAGAAGG
ATCTTATTCGGCTAGACCGGCAGATAAATCACATCGACAGATGAACACGACCGACAGATATGTATGTAAGAAGGATCTAATTCGGCTAGACCGG
CAGATAAATCAAATCGGCAGATGAACACGACCGACAGATATGTATGTAAGAAGGATCTAATTCGGCTAGACCGGCAGATAAATCAAATCGGCAG
ATGAACACGACCTTCAAAGATCCTCAAACTTATTGCACCCCACTGTATACAGTACTATAACAGGGTTATTAATAATATATGGATTATTAATGTG
CTTTAAGTACTTAAAGGAAGAGAAACCTGCGTTCTGCCCAACTATTACTCTATCGCCGGAAGTTTTAGTCCTAAGCAACGAACATATATTACCT
CACAAAGGCAAACACTTCTCCTTGCCGCACGTCTGATCCGTTGGTTAGACATACGGTAGATACGACCCAATTCAGAAAATATCGGAAAGACTCA
ATAGTTGTCTCCGCAAAGCCTGAACTTCAATACCGTACCTTCTCATGTTACCAACCATGAATCCCGCACTTGCAATGGTTTTGCTGTAAAGATT
AGATTCGAACCCCATTTGCTCGATTTCTAATTAAGCTTTTAGGTACTTAACTACTGCCTCGGCAGAAGACTGGGATCGGTGCCAATGTATGAAA
TACCCTGGCACGGGAACCCGCAATGACTGTGCCACGATCAAGGCCTGTGGCAGCGGTAAACACCGCGCTATATGTAAGAGGTTTTATGTTTATA
CGAGTGGCACTTGCTGACTTACTACAGCCATTTATAAAAATGGAGATGTATGGGTATGATATCGTCGGTCATTAATTAGGTGCGGCAACTTATT
GGATATTCTAGTGTGAAAAGACCGATGTGGCAATCCAAGGCCCCGAATTTCATCGAACCTGTTACGACCTTTTGCAAGACCCGAAACCTAACTC
CGAAGCCGATAGCTGTTGTATAAAGGGCAATAGAGCATCGGATGGATGCTTCAAGTAATACCCATCTCTTGGGTGTAAGGATTGGGATATTTAA
TTTAACTTAATGAATATTCTGGCTGGCGACCTTATGGCCGCTTAGGTTCCGTCTAGGAGCATAAGATACATTAAGCTATTTATATTAACGTACA
TGATAAGCGAGTGAAACAGACAAGCGAGTGGAACAAAAATCCCTAATCAAGCATGTAGACTAGAAATGTGCCGCATTTCCACTTCTGCACCAAT
CAAAATCTCCATATCCTTACCCCAAATTTCTCCCCTAGAAATGCGGCGCATTTCCATCTGGGCCTATCAACCATTCAGAAGTGATCTTGCAGGC
TAAATCATTTAGAATCCCATATAAAATAAAATGGCGCTATTGAAGTATGGGGGGCAAGCTCATTTAGCAAACAGAGGGGGCAAGCTCATTTGAA
CCCGCCAATTAAAATTGCCCAAAGTGGTAACGTAGACGATAAGCGGGTGTTACAGATAAGCGAGTGTTACAAAAGTCTTTATCTTAAAAATAAA
AATCGCAATAAAAACACAATAAAGTATCTAATCATTTAATTCTAATTGCTATACTCTTCTCCAGAGGCCTTAATACCCTCTTGACAGGTCCTTG
CATTATGCCCGGCCTTGCCGCATCTCCTACAGCGCCGAACCCCCGGTCGCACCGACCTTCCCTGACCACCACTCTTCGACAATTCGGCCACTAC
CTGCGTATCTATATCTATCTGATCAGTTAGATCCCGACCTTCCTCTACTGTCATCTTCCCTCTATTCTATAGGCGGGTTCTTTTTGCTCTGCGC
CGTCGGCTGAGTATCTCATTCG
```

Supplementary Figure 11 | Nucleotide sequence of the *SIX8*-*PSE1* locus of *Focn*Cong:1-1.

a, Representative read alignment results from cabbage infections at 10 dpi. Green arrows indicate coding sequences (CDS) of *SIX8* and *PSE1*. On the basis of RNA sequencing results, the untrancriptional intergenic region was deduced. **b,** Yellow and vivid blue highlights indicate the *SIX8* and *PSE1* CDS in opposite transcriptional orientation. Intron sequences are

highlighted in gray. Green highlight represents the miniature impala-like inverted repeat (mimp-IR) sequence. Red underline indicates the deduced untrancriptional intergenic region.

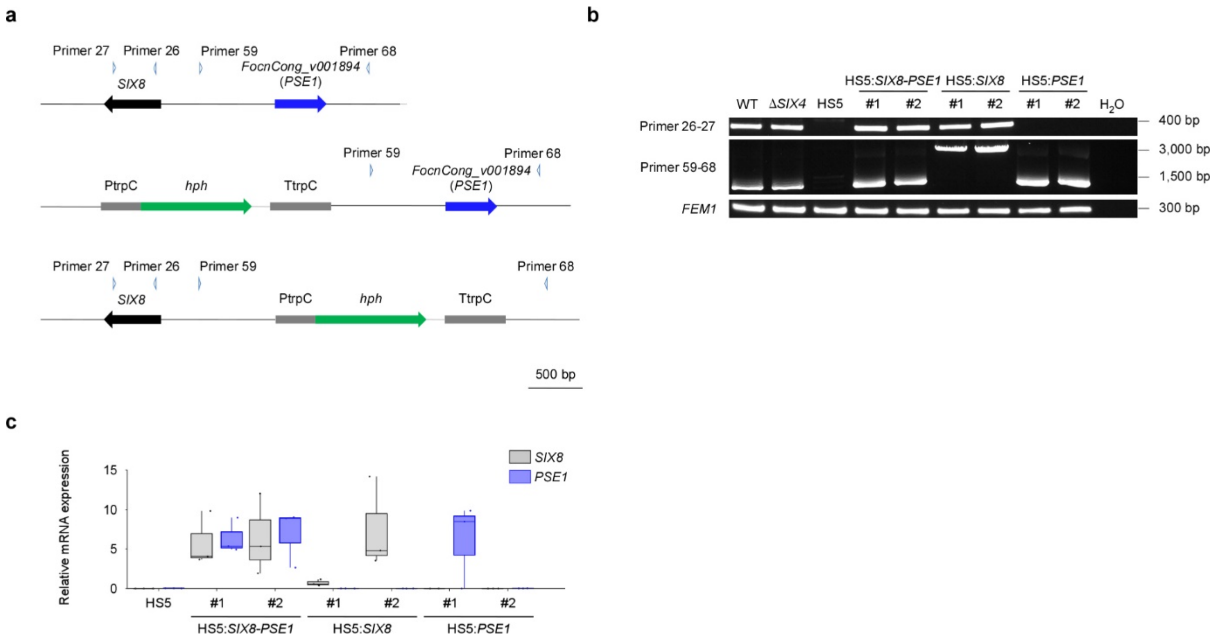

Supplementary Figure 12 | Introduction of the *SIX8-PSE1* locus, *SIX8* or *PSE1* into *Focn*Cong:1-1 HS5.

a, Schematic representation of the *SIX8-PSE1* locus (upper) and the loci in which *SIX8* (middle) or *PSE1* (lower) are replaced by a hygromycin B resistance gene (*hph*) cassette. Arrowheads indicate the primer locations for PCR verification. **b,** Confirmation of *Focn*Cong:1-1 HS5 transformants introduced with the *SIX8-PSE1* locus (HS5:*SIX8-PSE1*), *SIX8* (HS5:*SIX8*) or *PSE1* (HS5:*PSE1*) by PCR. *FEM1* was amplified as a control. DNAs of *Focn*Cong:1-1 WT, Δ*SIX4*, HS5, two independent HS5:*SIX8-PSE1* strains (#1, #2), HS5:*SIX8* strains (#1, #2) and HS5:*PSE1* strains (#1, #2) were used as a template. **c,** Relative transcript levels of *SIX8* and *PSE1* during infection of Arabidopsis *cyp79b2/cyp79b3* at 3 dpi. Data from three biologically independent samples are presented as fold changes compared with expression levels in *Focn*Cong:1-1 Δ*SIX4*. Expression levels were determined by qRT-PCR and normalized against *Focn*Cong:1-1 β-tubulin (*TUB2*). Boxplots indicate median value, estimated 25th and 75th percentiles, and whiskers represent 1.5 times the interquartile range.

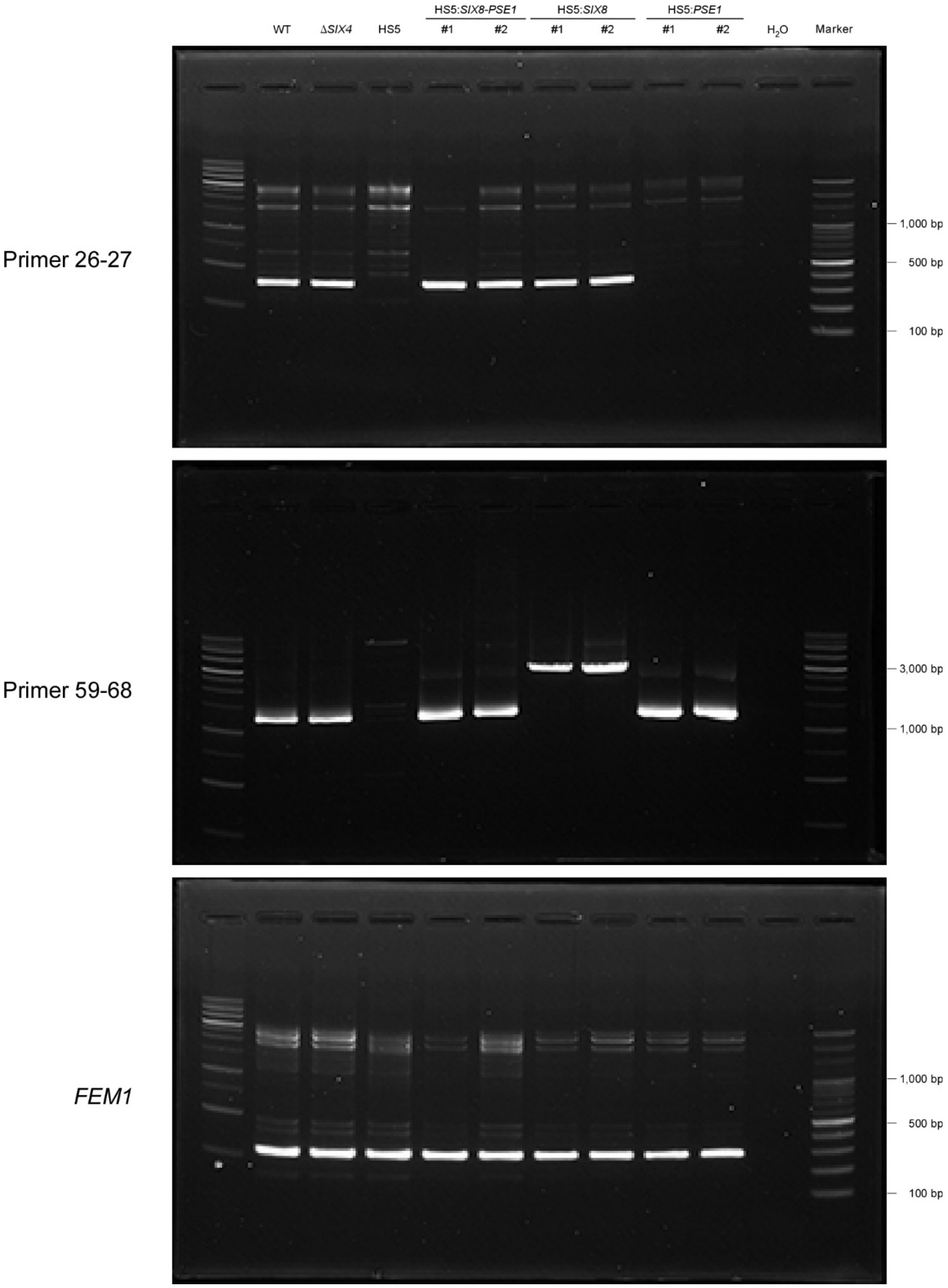

Supplementary Figure 13 | Uncropped gels from Supplementary Figure 12b.

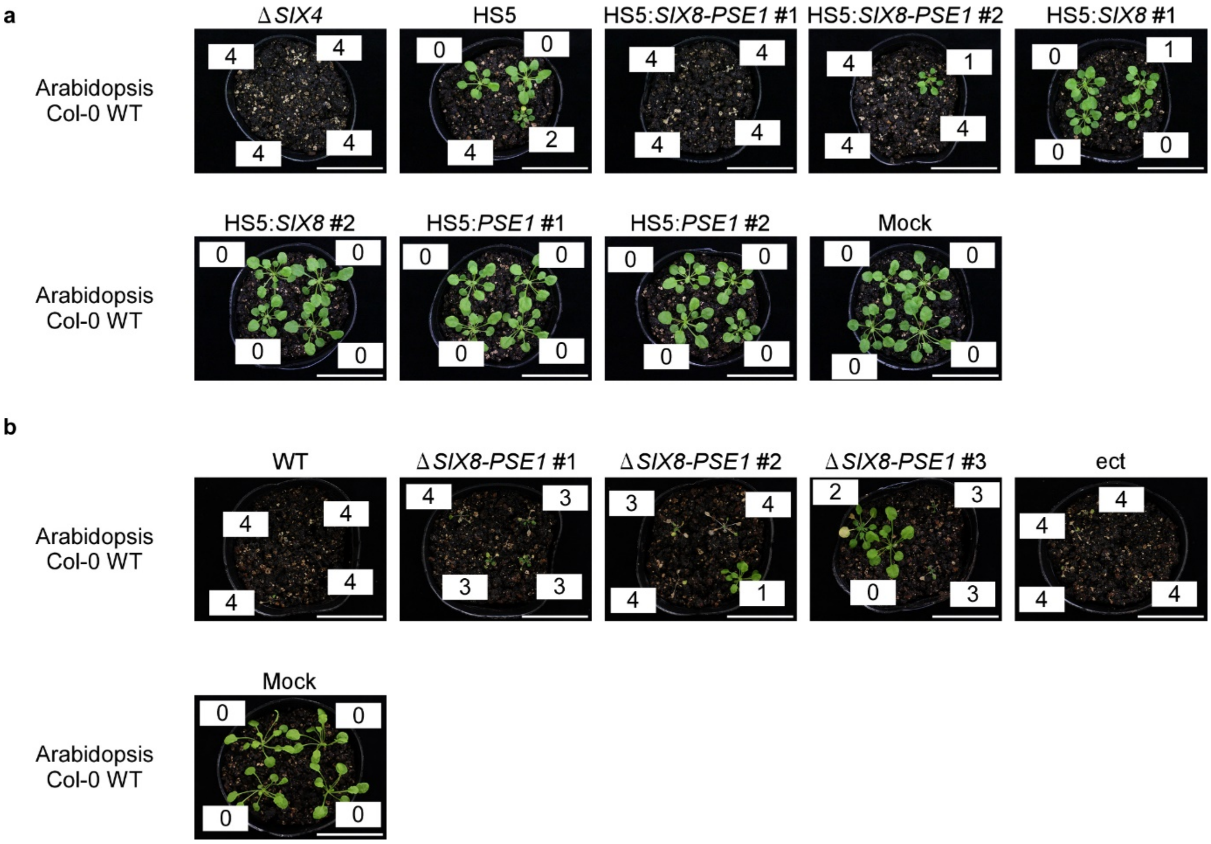

Supplementary Figure 14 | Representative images of infected plants from Figure 5c, d. Representative images of infected Arabidopsis Col-0 WT described in Figure 5c (**a**) and Figure 5d (**b**) with disease index scores. Scale bars indicate 3 cm.

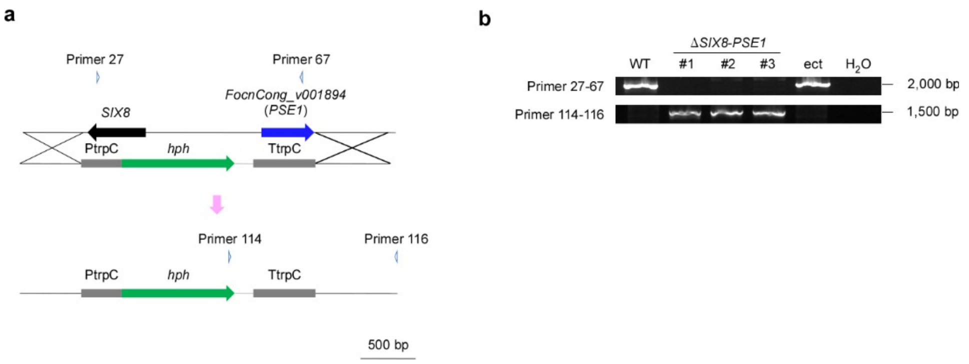

Supplementary Figure 15 | Deletion of the *SIX8-PSE1* locus in *Focn*Cong:1-1.

a, Schematic representation of a knockout of the *SIX8-PSE1* locus via replacement with a hygromycin B resistance gene (*hph*) cassette. Arrowheads indicate the primer locations for knockout verification. **b,** Confirmation of the *SIX8-PSE1* locus knockout by PCR. DNAs of *Focn*Cong:1-1 WT, three independent *SIX8-PSE1* knockout mutants (Δ*SIX8-PSE1* #1-3) and an ectopic transformant (ect) were used as a template.

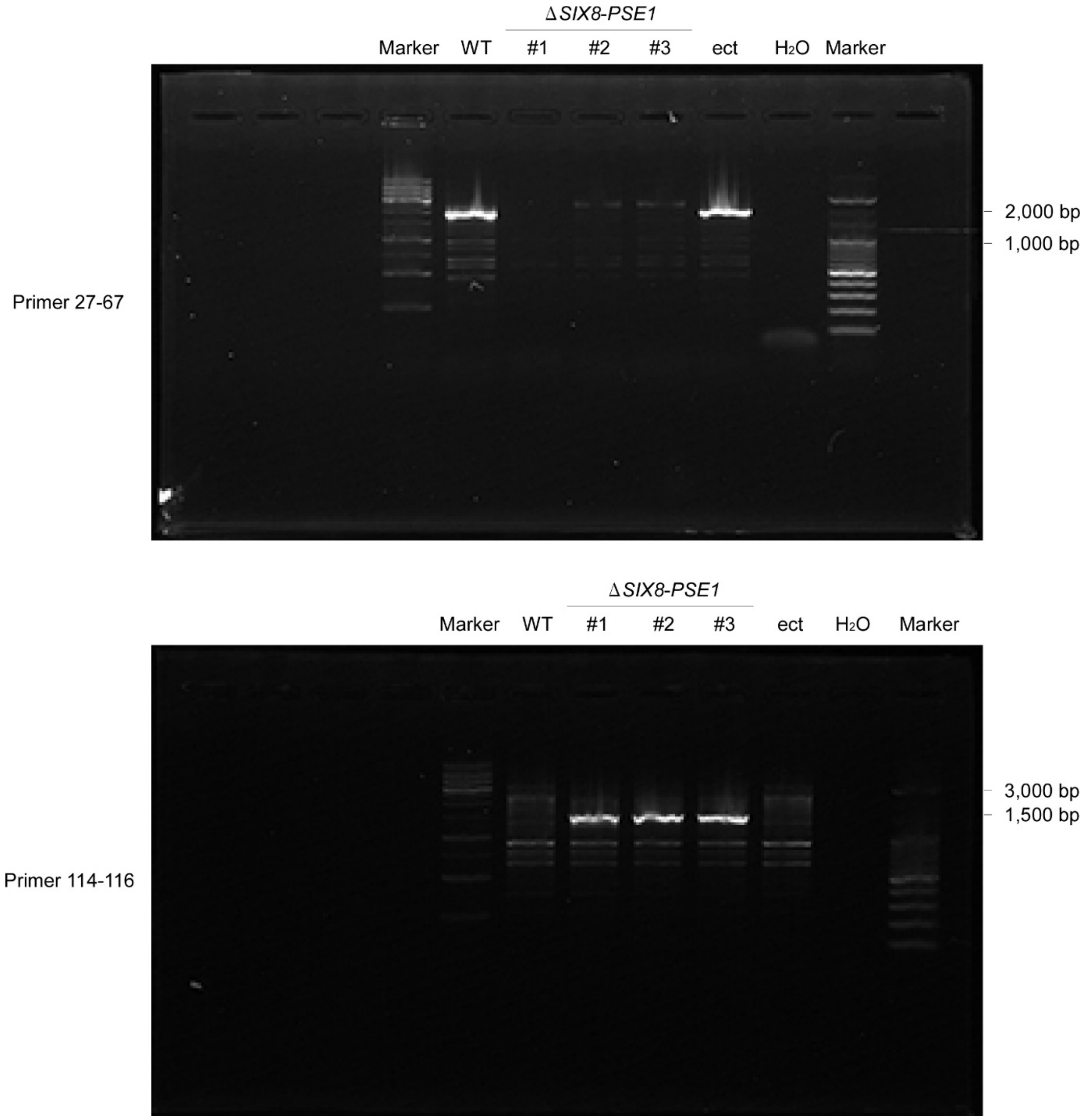

Supplementary Figure 16 | Uncropped gels from Supplementary Figure 15b.

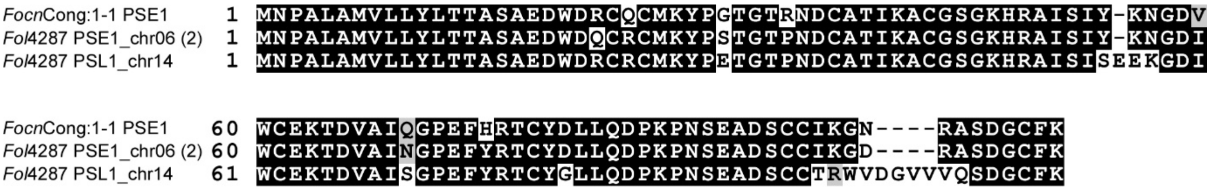

Supplementary Figure 17 | Alignment of the predicted amino acid sequences of PSE1 alleles.

Identical sequences are indicated in white on black, and similar amino acids in hydrophobic or hydrophilic features are highlighted in black on gray. Dashes indicate gaps introduced to maximize alignment. Multiple alignments of the amino acid sequences were made using the Clustal Omega method (https://www.ebi.ac.uk/Tools/msa/clustalo/).

>*Fol*4287_chr03:1040500-1048500
AACCCATCGCAATCGGCGTCGTTGATCGGGAGGATGTCGCTACCGATGCCACGGGCACCGTCCTCCGATATCTAAGGCGTTCTGGTTCTGAGGT
GCCGCCATTAGATCCGACAAAGGTGTTGTCAGAGAAGGTGAACTCTCCAAAATTCCTTCTCTGAAAACTGCTGTTAGGGAGCCGATGGCGTCCC
CGCAGTATTGTGTGGCCGAGTAGCCCGTAGAAAATCCCGCTCCTAACAGTGCACTAACAGTGCGTGCCAAGAAAGAATTTTTACAGGGGAGAGT
TGATCCACTACACCTACATCAACCAGGGTCGCCAATGTAGGGTAATTGATCACGTCGAGGGTTCAGGTTGATTGATGTAAGTGTATGAGTAATA
CACCAATCGATCAACCGGTGGGTTTCGGTGTAGTTGATCGGAACCTTGCGTGTTCCTTATCCTGTTATCCTGTAATCTGTATTTTTTCTTATCT
TGTTCTGTTTAACATGTTAACGGATTTCTTAACATGTTATACAGGTTGCCCATGTATGCTGTTCACCTCTGGCTGCGACCGCGTCAATTGACAA
AGTGGGTGACTCGGCGCGATTTAACGAGCTATCTGCCTCTGGGGGATCACCCAAAAGACTTGTTTCATACGACTCGATGCCCTGTGAGGGTGTA
ACCGGCGATGCTTCTGTCTGAAGAAATGTAGACTTGAAACCTGATGCTGTCGTGCGTTGTTTGCTTCCGAAGCCGTCTTCGAGCACGCTTGGTA
ACGACATCATGCTTAGCCTTGTCCTTTGGCGTGCCGTATTTTCGGGGCCGCCCGCGTTTCTTGGGTGTTCCCGGCATTGTGGGGATGATGGGTC
GTTGTGGGTACCGGCACGGTATGTTTTCCGTAGGATGGTATCAGGAAATGAGATTCCCCTGAACTGACAGCTAAATCCTGATTAGGTTAGTAAA
GTGAACGCGAAATTCTGGTTGGACAGAGGGAAATACACTAAGAGCCAAATTGGCTAGCTTACTGACCGCGAAATTCAAAATGCCAATTTTAGTT
AACCCGATGGCTTTGACTAGCGCCTCCAACACGGGGTATAGAATGATGTATATAATATCACGTGCATCCCATGATTGACCTAGCTACAGGGAAA
AGAGTCTCTTTTTTTTTCAAGGGAAGGCAATTTTCACCAAGTCCCAGCTTTTCCCCTATTGAAATCCTTAGACATAGCTGCTAACCCTGACCCG
GATGGGACTGCCTATGTGCCACTGTTGGAAAACGAGACAGTACTTGCACCTTGTGTAAAAATGTGCACCACCCCACCCGGCAAGACTCGCACCA
CTTCAGTTACCGCCCGTGCCAGCGCTCCCCCGCTCAAGGTAACAAGCACGTTCGACGTCCTTCCACCAAAAGTTCTGTCCACCCTTACCGCCAG
AGTGCGGGGGTCATTACGAAATGCGGAGAGATAGCATCCTGTATATGCCTGAAACGCCGTGCCAGCACCCGTTATGCAGGCGAGTAAAATGCCA
CGGGTGTCGCTGTAATTTAAATTAGACCAACCCTAAAATCTATATCAAGCTACTTACCGTTCAACAAGGGCTCTCTCGTCAAGAGAATGAGGTT
CATTAATAGCTTCTTCAATAGTTGCGGCTTGGTCGTCCCTTGATGTACTGATAAGTGTGACGGACGTATTCATGAGAATCAGACATACCTCTGC
TTAATATGAGTAGTAGCCACAGAGACGGCTAGGGGGAGGAGTAAAAGCATGCGTGAGGGATGCATAGTTGCCGTTTATGGTATTGAGGAGGGTT
TATAAGGTCGCGAAGACTTATTTAAGAGGCGTAACTAAGGGTGGGTAAACCCGGACTCTTGACGCAGTATGAAAGGATGTCCGGGAACGAGAGA
ATAGAGGGAGAGTCTAACATTAAATATGTTTCTAGTCTTATAAGGCCCACTTCCCCGCTTAAATCTCTCTTAAAAGCTTTACTTTTAGTACAGG
TAGAGTATAGAGTTTAATTCCCTTATAACATAATTTAAAAGCTCCATTCTCTGTTATTACAGGGTACATTTTAAAGTTTATTAAAAAATAAAGT
TTAACTAACGTGTTTGATAAGTGAGTGGGTCATCCAAGTGAGTGGGTCAATCTTATTTCCACCTTAAATCCGGGGTTTAAAAAGTTCTTTAAAA
TTTCAAACTAAGCCTAAATTGGCTGTAATTTTAGTAGCCTATATATAAGTCTTAAAATATATCATATAGATTTTTTAAGATTTTTAGAAAAATT
CTATATAGCTATAAACCTTAAAACCTATTTATACTGTATTTTATACTGTATAATTAAGCTTTACTATATAAAATAAAATAGCTAAAAATCTTAA
AAAAATATTTTATAATAAGGTTAATTATACTTTTCTTAAAGATGTTTTTTTTAAATTTTTATCTATTTTATTTAGAGTAAAAAGATACTATAGG
GTAGGTTTTTTAATACTATACAGTGCAGTGACTCACTTACTTGTCTGACCCACTCACTTATCAAACACGTTATACCATATACCTGTCGGTTGTA
TTATTAAAGATAATTCTGCCGCTTGAACTTAAATACTAGTATATAAGACCGGCAGAAGAATTCGGCAGATTTATAATAAGAATCGGATCTGGCT
AGGCCGACTACTCCTACTGTGGTAAAAATAAAAGCTTAAACTATAAGCCGAAGTACAGTGGTGTGCAATAAGTTTGAATCCAAGATGAAGTATC
TGCCTTTTCGCTCTAGCCCTGTCAGAAGCCTGACTAGCTATATCAGGTAGATATCTACAGTGGGGTGCAATAAGTTTGAGGATCTTTGAAGGTA
CCGAAGCAGCCCGTCTAGCCGGATTAGATCCTTGTTACATATCTGTCGGTCGTGTTCATCTGCCGATTTTATTTATCTGCCGGTCTTATGTACT
AGTATTTAGGTTAAAGCGGCAGAACTACCTTTGACTTGTACTGTATTCTTTTAGTGTTTCGGCATATAGTTTAAGCTTTCATTCATATTACAGT
GGGATGCAATACAGTAGGATGCAATAAGTTTGAATACCGAGCGTGTATCTTCTTTTGCCGCTAGCCTCTTTGAGACAGCTACCACCTGCATATC
AGTTGTTGCACGGCGAATAAGTATATACTGATTGGCTAGATCGAGCTTCTTCCTCAGATTAGAAGCAGGCTGAACTCCAAATAGGCTAGACAGG
GGGTATCAGTACTTCAAAAGGTATTCAAACTTATTGCATCCCACTGTAAGTTTTGAATCCAGACTGAAGTATCCGTAGTTCTGCCGCTTCAACC
TAAATACTAGTACATAAGACCGGCAGATAAATAAAATCGGCAGATGAACACGACCGACAGATATGTAACAAGGATCTAATCCGGCTAGACGGGC
TGCTTCGGTACCTTCAGAGATCCTCAAACTTATTTGTTGAATATTGGAGGGTTCACCCTCAACTTCAGGATTAAGGTTCAGGATTAAAGTTTGG
AGTCCTAAACTTAGATCGTAGGACTTTCATCTAGGACTTTCTAGGACTCAAGTCCTAGAAGGAACCAGTCGAAGATATAAGGTCTCAATAGTCC
TTCGATTGCGACAGAATGAAGAGACACAATTCACCTTAATATCTGACATTATTGCTCCCCACTGTAATATGAATGAAAGCTTAAACTATATGCC
GAAACACTAAAAGAATACAGGTCAAAGGTAGTTCTGCCGCTTTAACCTAAATACTAGTACATACAGTGGGATGCAAAAAGTATTCGCAGGTGTG
AAAGTCGCGTTGATCGGCCCTAGCTTCTCTAGTTAGTAGCTTAGCTTAGCTTAGGTACTTGATACTATGTAACCCCCCTGATTTAGGTGTAATA
TAAGGTATTGAGGACCTGACAAGCTACCCCTGANNNNNNNNNNNNNNNNNNNNNNNNNNNNNNNNNNNNNNNNNNNNNNNNNNNNNNNNNNNNNNN
NNNNNNNNNNNNNNNNNNNNNNNNNNNNNNNNNNNNNNNNNNNNNNNNNNNNNNNNNNNNNNNNNNNNNNNNNNNNNNNNNNNNNNTTTTTGCATCC
CACTGTAAGACCGGCAGATAAATCAAATCGGCAGATGAACACGACCTTCAAAGATCCTCAAACTTATTGCACCACACGGTATACTATAACAGGG
TTATTAATAATATATGGATTATCAATGTGCTTTAAGTACTTAAAGGAAGAGAAACCTGCGTTCTGCCCAACTATTACTCTATCGCTGGAGTTTA
ATCCTAAGCGACGAACATATATTACCTCACAAAGGGAAACACTTCTCCTTGCCGCACGTCTGATCCGTTGGTTAGATATACGGTAGATACGACC
CAGTTCAGAAAATATCGGAAAGACTCGATAGTTATCTCCGTGAAGCCTGAACTTCAATACGCTACCTTCCCGTGTTACCAACCATGAATCCCGC
ACTTGCAATGGTTTTGCTGTAAAGAGTAGATTCGAACCCCATTTGCTCGATTTCTAATTAAGCTTTTAGGTACTTAACTACTGCCTCTAGTGAT
GCAATTTCTTGTACACGTACGTACGTACGTACTGTATTTTAGCCGCTGTACGTACGTACAACCTTATTGTACGTACAAATATGTACCATACAAA
TGTCCCCCTTCCTTTTTAGGTAATACTGGTAACTTAGTAGGCTTCTCACGGTCCTCGTCTTCTGCCATTTTGGCCTCAAACCTCTGATTCCACC
TCAGTGCCAACCCCCAATATATCACCCGTCGACTGTAATACACCCTCCCACTCAAAATCTCCACTGGTGGAGGCCATCACACCATCCATCCAGA
CCTTCTTGCCCTGCGCATTCACAAATAACTCGAGATATGAATCTTCGAGTCCTAGCCAGTCCTTCTCCTCAAGATCCGCCCAGCGCTTTTTATG
GGCGTACTTCTGATCCTTAAGGCGATCTAATACGCGGTCGTTCATAAAAATGAAAGCCTGCATGTCAGCGTGCATGGGCGTAAGGCGATTTCGA
GCTTTCGTGTGTATGAAATTAATAGCAGAGAAACTTCGTTCTGAGGGCACAGAGTTCGCAAGCGTGTTCATTACCTTCACGGCTACTCTAGCCA
GAATCGAGCCCTGGTTGAGGAGGCACTGCCACGCCATTGCTGGCTTGAAGCCATCATCGTATACAGCGCTGTGCTTATTAAACAAGCCGTCTGG
GCCGCCTGTACGCATTCGGAAGTGTGTGAATTCGGAAAGGGCACGGTGATATTCGTCGTCGTTTTTCAGCTGTTTCTGCATATACTCTTGTACG
CGCGCAAAGACGCTAGGCGGCAGTTTCGAATTTGGACCTGTTGTATCAGGCCGGAGGGGCATCGGCGATCCAATGTATATCGTACGTCTGCTTA
TCGAGCCGGGCTTTGAATATAGCGTCAATGTCGTCCCACGGAATATCAGGAAACTGATCAGCCTCTCCCATCTCAGACCATTTGCTTTTGATTT
GCAGCCAGCGATTTACAACGTGTGCGATTCCCGCCCCATCAGCCTCGGAAGCGTGTTGAAATTCGTTCACGGGTTTTAGCACAGCGATCGCAGC
CTCCAGTTTTAGCCAGAAGCTGTTGTCTTTGATGATTCGAACCGCTTCGGGCAGGAGAACGGGGCAATCTTGAGATCGCAGCTCATCCCGTACG
TCTTTTCGAATAGACCAGTCTCTGGCAGGGTCTCGAGAACGGAGGAGGGAGAAGAAAGAATTGTACTGAGAACCCCAGCGTGTAATTATGCTAT
TCGGAACGAGCGAGTTAGTATATCGATTTCTCAGTCACAGCTAACGATTTACCGTTACCTTCTGATCAGCGCGCGAGTTACTCCGTTCCACCTT
GTGCGTTGGCACATTCTCAATCTTTGCAATTGTAATTTAGACCGTGAGAAGAACTTGAGAAGGGTGCTGACGTTATTACAGAGATCCTCAAAGA
ATGGAAGAAGAAGGATATCTTTAATTAGAAGCTGAAGGCCGTGAGAATCGCATAGAGAGAAGTTGATATGTGAGAGCTCTGGGAATCTCCCTAG
CAAGCCGTGTACAGAGCGCATCGTGTTTTCGGTATCTGTACAGATAGAATTGATTCTGGAGAGATCACCGCCGCACAGTTGCTGCATTTCTCCC
CATATCAGCTTCACCCAATTCTCCGCTGTGTGCTGTTCGTCTCCCGTGTCAAACGTTTTCCAGTAGAAGGCTGGGCCGTCTGGTAGCTGTACCG
AGATATTAACGATTCTGTGACCCGAGACATTATCTGAAGCGTCAAATATGATGTTGAGCCATTCAAAGCTGGTAATGCGTTTGAAAACCTCGTC
ATAGAGTTCCTGATAGAAGTCGGGAAGAATTCTCGTGATGGCGGCGCGGCTAGGAGGCTTCCAGCCAGGCTTAATGCGAGTGAAGAAATAACGC

CAACGGCGGCTCTCGAAGAGGCTGAAGGGCCGACCACCGGCGATTACAGCAGCGGCGGCATCATAGGCATAACTGTGACTCTCGTCCTGCGAGA
TATGCTGAATTCCGACCTCGGTTATCTTCTTTTGTATGGCTTCTGAGTAGGAGGGAAGATAGCCAGCGTTATCTTGAGTTTGACGCTCTTGTCG
CTGCTTGGCTTGCCATTTCTCACACGCAGCGAGATGCTCTTTCGGTCTCCCAACAGAGGTCGCTGCAAAATCTTTTTGGCAGTGACGGCATCTG
TATCGCTTGCTTTTCTTGGAGTTAGAGCGGCCGAGGTCGATAAACTGTCGCCAAACGAAGGCAGCGTTAGCTTGGGTGGGCGCCATCTTTTTGG
CGATACCTTCAATCGGGAGAGAATGGCAGAGTGTGTTGAGCTGCTCAGCCAACGGAGTTTTTGTACAGCCCCCCGTATTTGTATAATATGACCC
ACAAATTCCCCTATTAGGATTATTATGGAACCCATTGTACGTACATAGTACGTGTACGTACTTTTTCTTTCTGTACGTACGTACGTACAACACG
GTTGTACATACTGTACAACATCACTAGGCAGACTGCCTCGGCAGAAGACTGGGACCAGTGCCGATGTATGAAATACCCTAGCACGGGAACCCCC
AATGACTGTGCCACGATCAAGGCCTGCGGCAGTGGTAAACACCGCGCGATATGTAAGAGGTTTTATGTTTATACGAGTGGCACTTGCTGACTTA
CTATAGCCATCTACAAAAATGGGGATATATGGGTATGATATCGTCGGTCAACAATTAGGTGCGGCAACTTATTGGATATTCTAGTGTGAAAAGA
CCGATGTGGCAATCAATGGCCCCGAATTTTATCGAACCTGTTACGACCTTCTGCAAGACCCGAAACCTAACTCCGAAGCCGATAGCTGTTGTAT
AAAGGGCGACAGAGCATCGGATGGATGCTTCAAGTAATACCCATCTCTTGGGTGTAAGGGTTGGGATTTTAATTTAACTTAATGGATATTCTGG
CTGGCAACCATATGGCCGCTTAGGTTCCCTCTAGGAGCATAAGATACATTAAGCTATTTTAACCTACATGATAAGCGAGTGAAACAGACAAGCG
AGTGGAACAAAAATCCCTCAACCTACATCATTTCTAGTTGATCAATTGATTCTCAACCACCCAATATGTGACAGTTAAATAAAGAAGCTGATAT
CCTGCTTGCTATTTAAGCTCTTCAAAATGACCCAAAACCAAGTCTCCGACGCGCCGCAAAAATCTACAAAGTTAGCCTTACAACCCTTTGGCGT
CGGCAGCAAGGCATTCTTTCCCAGCGCGACACTATCCCGAAATCACGCAAACTATCTGATCTAGAAGAACAGATAATGAGCCAAAATGGCTGGA
CAAATAACGAACTGGGCCTTGAGTGGCTAAAGTACTTTAATCGGTCTACAACTAACCGATCAACAGGCTGGCGATCCATTCCACGTCCACCGTC
CACGTATCCACGGCGTGGAAATGGATGGTCCGTGCATCCATTTCCATCCATTCCATGGAGGGCGCCCGCTACACGCGCGCCCGGATGCGCATTT
AGATTTCTTT

Supplementary Figure 18 | Nucleotide sequence of the *Fol*4287 *SIX8-PSE1* locus with TE inserted.

Yellow and vivid blue highlights indicate the *SIX8b* and *PSE1* coding sequences (CDS) in opposite transcriptional orientations. Intron sequences are highlighted in gray. Red underline represents the sequence encoding a putative transposase gene.

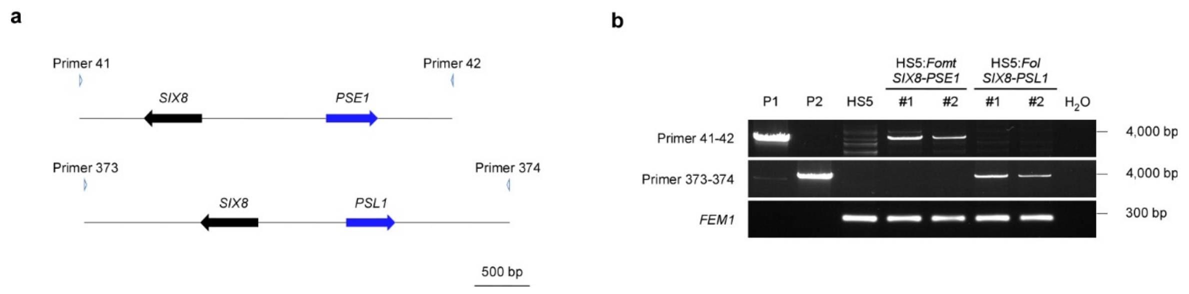

Supplementary Figure 19 | Introduction of the *Fomt SIX8-PSE1* locus and the *Fol SIX8-PSL1* locus into *Focn*Cong:1-1 HS5.

a, Schematic representation of the *Fomt SIX8-PSE1* locus (upper) and the *Fol SIX8-PSL1* locus (lower). Arrowheads indicate the primer locations for PCR verification of transformation. **b,** Confirmation of *Focn*Cong:1-1 HS5 transformants introduced with the *Fomt SIX8-PSE1* locus (HS5:*Fomt SIX8-PSE1*) or the *Fol SIX8-PSL1* locus (HS5:*Fol SIX8-PSL1*) by PCR. *FEM1* was amplified as a control. Plasmid DNA containing the *Fomt SIX8-PSE1* locus (P1) or the *Fol SIX8-PSL1* locus (P2) and DNAs of *Focn*Cong:1-1 HS5, two independent HS5:*Fomt SIX8-PSE1* strains (#1, #2) and HS5:*Fol SIX8-PSL1* strains (#1, #2) were used as a template.

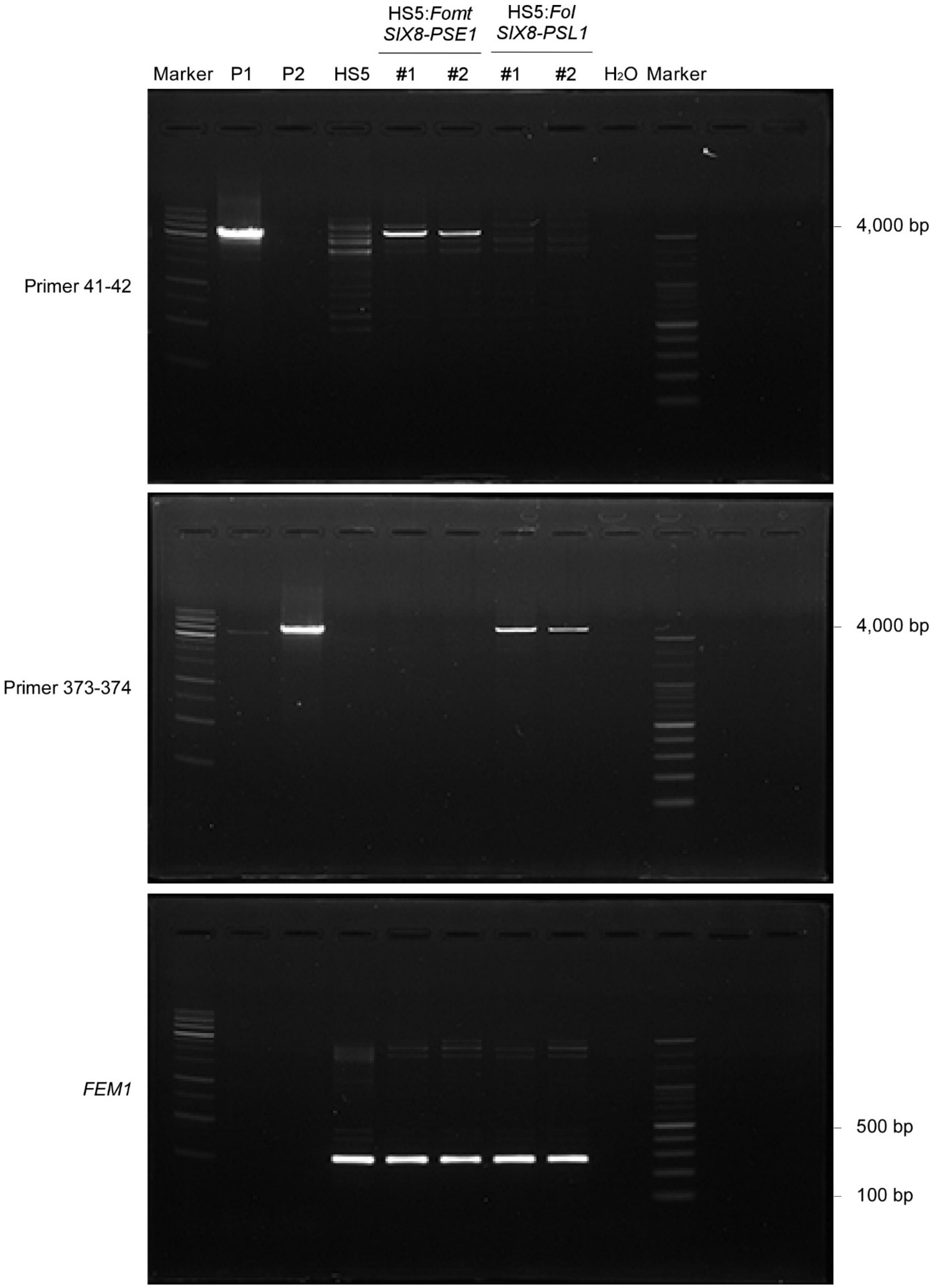

Supplementary Figure 20 | Uncropped gels from Supplementary Figure 19b

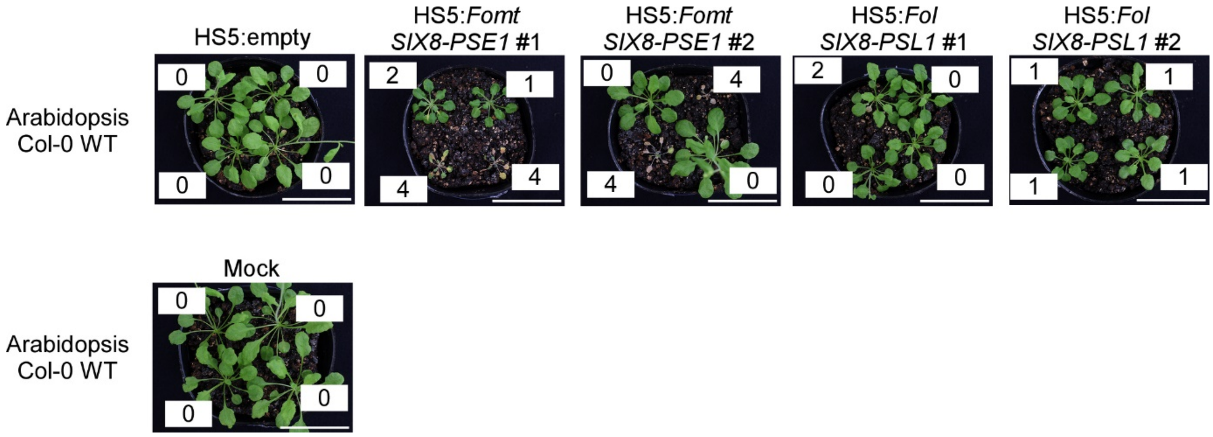

Supplementary Figure 21 | Representative images of infected plants from Figure 6c. Representative images of infected Arabidopsis Col-0 WT described in Figure 6c with disease index scores. Scale bars indicate 3 cm.

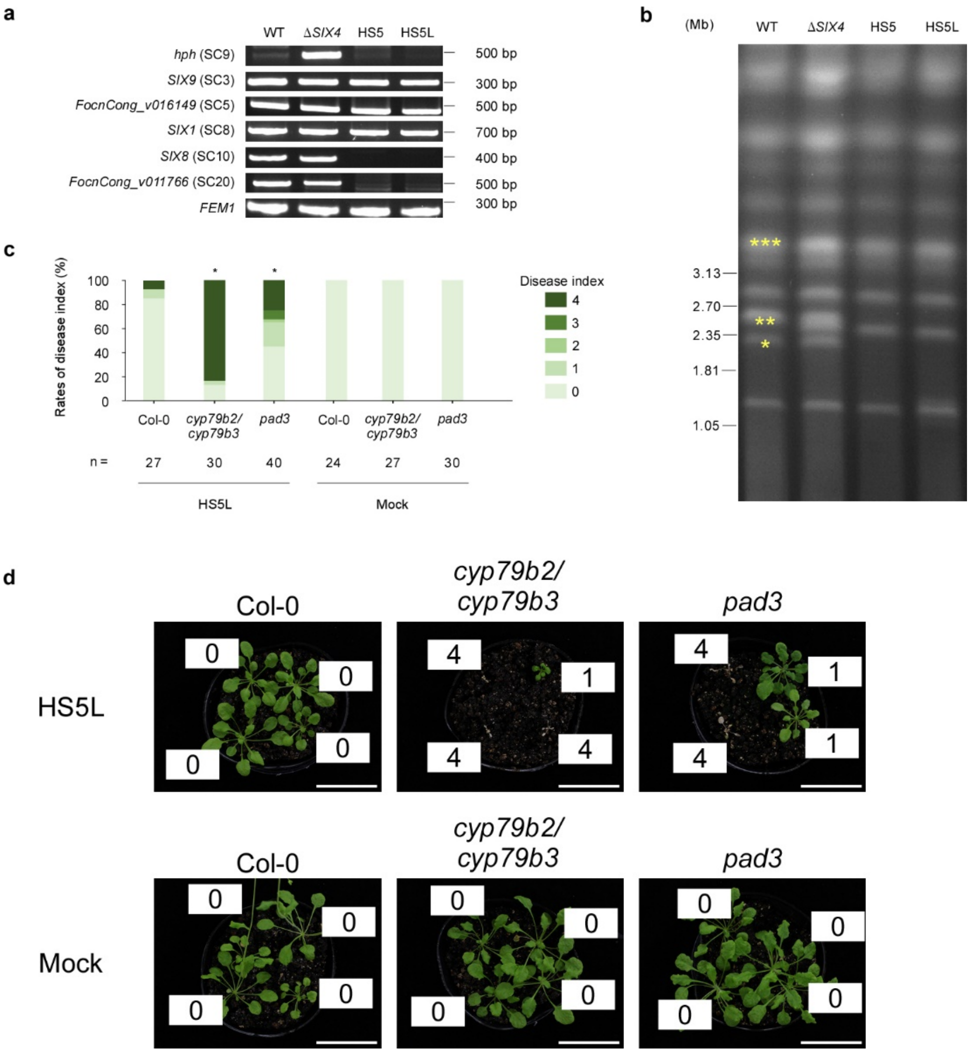

Supplementary Figure 22 | Virulence of *Focn*Cong:1-1 HS5L on Arabidopsis.

a, Detection patterns of genes located on particular scaffolds (SCs) in *Focn*Cong:1-1 WT, Δ*SIX4*, HS5 and HS5L. Hygromycin B resistance gene (*hph*), *SIX9*, *FocnCong_v016149*, *SIX1*, *SIX8* and *FocnCong_v011766* were detected by PCR. *hph*, *SIX9*, *FocnCong_v016149*, *SIX1*, *SIX8* and *FocnCong_v011766* are located on SC9, SC3, SC5, SC8, SC10 and SC20, respectively. *FEM1* was amplified as a control. DNAs of *Focn*Cong:1-1 WT, Δ*SIX4*, HS5 and HS5L were used as a template. **b,** Electrophoretic karyotype of *Focn*Cong:1-1 WT, Δ*SIX4*, HS5 and HS5L. Asterisks indicate chromosomes on which *SIX* genes are located as follows: **SIX4*, ***SIX8*, ****SIX1*. **c,** Disease index of Arabidopsis Col-0 WT, *cyp79b2/cyp79b3* and *pad3* challenged with *Focn*Cong:1-1 HS5L or water (mock) at 28 dpi was scored as described in Methods. n denotes the number of plants investigated. Asterisks represent significant difference

from Arabidopsis Col-0 WT infected with *Focn*Cong:1-1 HS5L (*$p < 0.01$, Mann–Whitney U-test). **d,** Representative images of infected plants described in (**c**) with disease index scores. Scale bars indicate 3 cm.

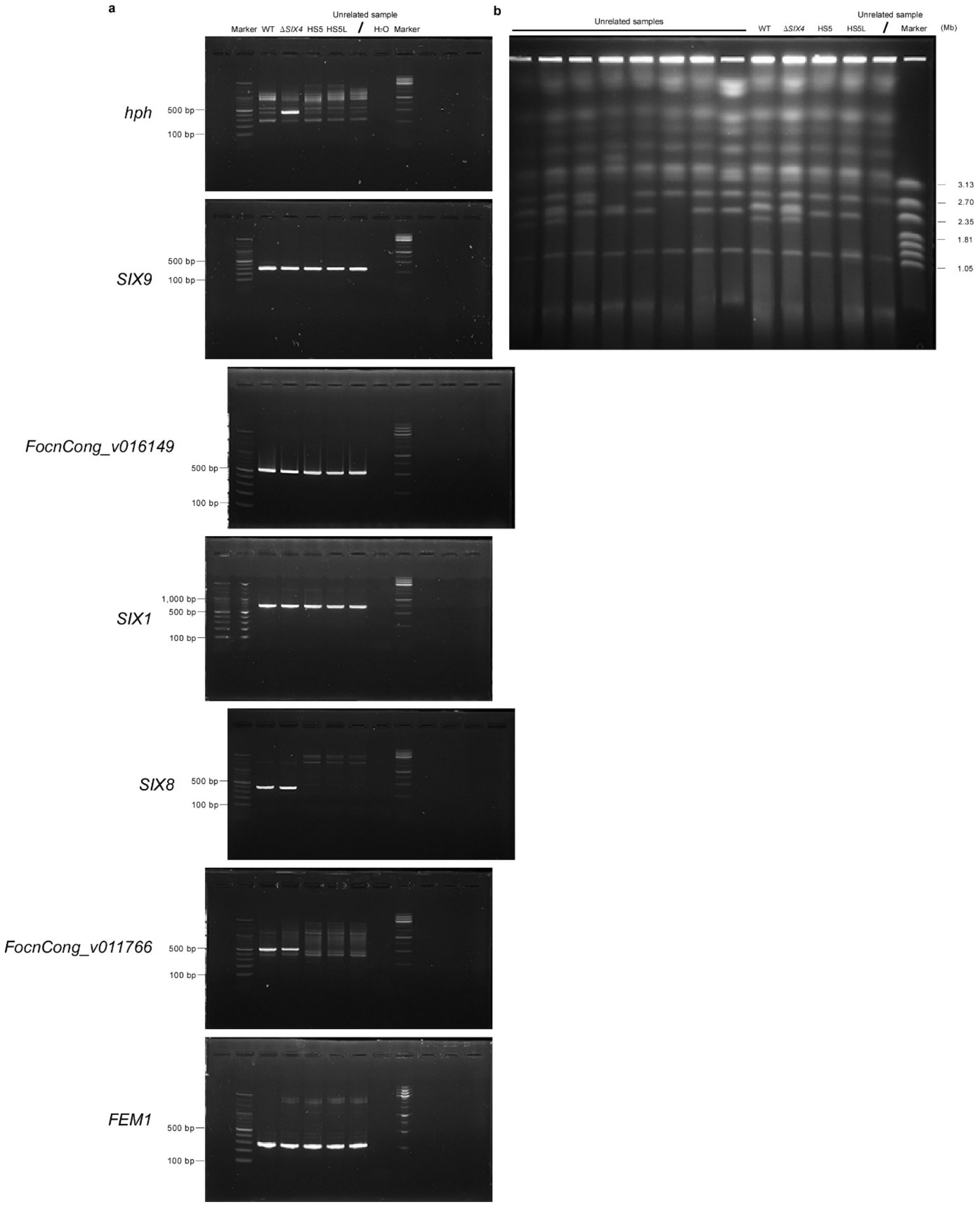

Supplementary Figure 23 | Uncropped gels from Supplementary Figure 22.

a, Uncropped gels from Supplementary Figure 22a. **b,** An uncropped gel from Supplementary Figure 22b.

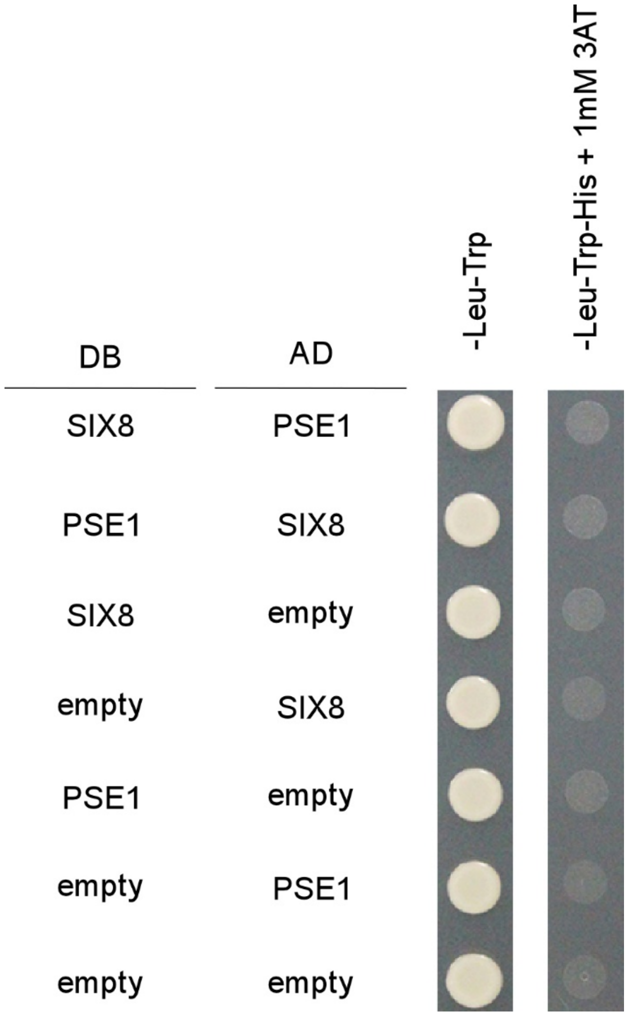

Supplementary Figure 24 | Yeast two-hybrid assay indicates no direct interaction between SIX8 and PSE1.

SIX8 and *PSE1* were cloned into bait vector (pDEST-DB; DB) and prey vector (pDEST-AD; AD). DB and AD were transformed into yeast strain *MAT*α Y8930 and *MAT*a Y8800, respectively. After mating of the yeast transformants, yeast suspension was spotted on synthetic defined (SD) medium lacking leucine and tryptophan (-Leu-Trp) for selection of diploid cells (left) and on SD medium lacking leucine, tryptophan and histidine and supplemented with 1 mM 3-amino-1,2,4-triazole (-Leu-Trp-His + 1mM 3AT) for verifying a protein-protein interaction (right).

Supplementary Table 1. Information for genome assembly of *F. oxysporum* isolates.

Organism	Isolate	No. of scaffolds/contigs	GenBank assembly accession	Reference
F. oxysporum f. sp. *conglutinans*	Cong:1-1	22 + 125[a]	RSAI00000000	this study
F. oxysporum	Fo5176	19	GCA_000222805.1	Fokkens et al. 2020[1]
F. oxysporum f. sp. *matthiolae*	PHW726	66 + 517[b]	GCA_009755825.1	Yu et al. 2020[2]
F. oxysporum f. sp. *lycopersici*	4287	15 + 73[c]	GCA_000149955.2	Ma et al. 2010[3]
F. oxysporum f. sp. *cubense*	TR4	15	GCA_007994515.1	Warmington et al. 2019[4]
F. oxysporum f. sp. *cubense*	160527	12	GCA_005930515.1	Asai et al. 2019[5]
F. oxysporum f. sp. *radicis-cucumerinum*	*Forc* 016	33	GCA_001702695.2	van Dam et al. 2017[6]
F. oxysporum f. sp. *melonis*	26406	96	GCA_002318975.1	van Dam et al. 2017[6]
F. oxysporum f. sp. *cepae*	FoC_Fus2	34	GCA_003615085.1	Armitage et al. 2018[7]
F. oxysporum f. sp. *vasinfectum*	TF1	17	GCA_009602505.1	Seo et al. 2020[8]

[a]Number of scaffolds + non-scaffolded contigs.

[b]Number of core scaffolds + scaffolds.

[c]Number of chromosomes + non-scaffolded contigs.

Supplementary Table 2. *F. oxysporum* strains used in this study.

Strain	Genotype	Reference
Focn Cong:1-1	wild type	Kawabe et al. 2005[9]
Δ*SIX4*	Δ*SIX4*::*hph*	Kashiwa et al. 2013[10]
HS1	ΔSC9	this study
HS2	ΔSC3/SC9	this study
HS3	ΔSC3/SC9/chr$^{SC10/SC20}$	this study
HS4	ΔSC3/SC9/chr$^{SC10/SC20}$/chr$^{SC16/SC18}$	this study
HS5	ΔSC9/chr$^{SC10/SC20}$	this study
HS6	ΔSC5/SC8/SC9/chr$^{SC10/SC20}$	this study
HS6-BLE	ΔSC5/SC8/SC9/chr$^{SC10/SC20}$::*ble*	this study
HCT1	ΔSC5/SC8/SC9/chr$^{SC10/SC20}$::SC8/SC9/chr$^{SC10/SC20}$/*hph*/*ble*	this study
HCT2	ΔSC5/SC8/SC9/chr$^{SC10/SC20}$::SC8/SC9/*hph*/*ble*	this study
HCT3	ΔSC5/SC8/SC9/chr$^{SC10/SC20}$::SC8/SC9/*hph*/*ble*	this study
HCT4	ΔSC5/SC8/SC9/chr$^{SC10/SC20}$::SC8/SC9/*hph*/*ble*	this study
Δ*SIX4*-GFP	Δ*SIX4*::*hph*/*nptII*/*GFP*	this study
HS2-GFP	ΔSC3/SC9::*hph*/*GFP*	this study
HS5-GFP	ΔSC9/chr$^{SC10/SC20}$::*hph*/*GFP*	this study
HS5:*SIX8-PSE1*#1	ΔSC9/chr$^{SC10/SC20}$::*SIX8-PSE1* locus/*hph*	this study
HS5:*SIX8-PSE1*#2	ΔSC9/chr$^{SC10/SC20}$::*SIX8-PSE1* locus/*hph*	this study
HS5:*SIX8*#1	ΔSC9/chr$^{SC10/SC20}$::*SIX8*/*hph*	this study
HS5:SIX8#2	ΔSC9/chr$^{SC10/SC20}$::*SIX8*/*hph*	this study
HS5:*PSE1*#1	ΔSC9/chr$^{SC10/SC20}$::*PSE1*/*hph*	this study
HS5:*PSE1*#2	ΔSC9/chr$^{SC10/SC20}$::*PSE1*/*hph*	this study
Δ*SIX8-PSE1*#1	Δ*SIX8-PSE1* locus::*hph*	this study
Δ*SIX8-PSE1*#2	Δ*SIX8-PSE1* locus::*hph*	this study
Δ*SIX8-PSE1*#3	Δ*SIX8-PSE1* locus::*hph*	this study
ect	*hph*	this study
HS5:*Fomt SIX8-PSE1*#1	ΔSC9/chr$^{SC10/SC20}$::*FomtSIX8-PSE1* locus/*hph*	this study
HS5:*Fomt SIX8-PSE1*#2	ΔSC9/chr$^{SC10/SC20}$::*FomtSIX8-PSE1* locus/*hph*	this study
HS5:*Fol SIX8-PSE1*#1	ΔSC9/chr$^{SC10/SC20}$::*FolSIX8-PSL1* locus/*hph*	this study
HS5:*Fol SIX8-PSE1*#2	ΔSC9/chr$^{SC10/SC20}$::*FolSIX8-PSL1* locus/*hph*	this study
HS5:empty	ΔSC9/chr$^{SC10/SC20}$::*hph*	this study
HS5L	ΔSC9/chr$^{SC10/SC20}$	this study

Supplementary Table 3. Primers used in this study.

Primers	Sequences 5'-3'	Purpose	Reference
SIX1F	CTCTCAATCCTTGGGTTTGG	Detection of *SIX1*	this study
SIX1R	TCCCCTCTCCACTCGAATAA	Detection of *SIX1*	this study
SIX8F	GTGGCTGCTACGCATATTGA	Detection of *SIX8*	this study
SIX8R	TCGTGTACCGCTTGTGAGAG	Detection of *SIX8*	this study
SIX9F	GCCGAAGCCCAGAATAAGA	Detection of *SIX9*	this study
SIX9R	GGTGCACTGTCCCATACTGA	Detection of *SIX9*	this study
32	ATGGGATTGATTGCTTGTACT	Detection of *FocnCong_v016149*	this study
33	TGCTTGCTCGAATAGGCT	Detection of *FocnCong_v016149*	this study
18	ATGGTACAGGGCCAATG	Detection of *FocnCong_v011766*	this study
19	TCAAGACTTATTACGCTTCTCTTG	Detection of *FocnCong_v011766*	this study
A5'	TCTCGATGAGCTGATGCTTTGG	Detection of *hph*	Zhang et al. 2000[11]
B3'	AGTACTTCTACACAGCCATCGG	Detection of *hph*	Zhang et al. 2000[11]
3655	ATGACCGAGATCGGCGAGCA	Detection of *ble*	this study
3656	ACATGCAATTATCTTTGCGAACCC	Detection of *ble*	this study
59	CATAAGACCGGCAGATAAATCA	Detection of *PSE1*	this study
68	TTTTAATTGGCGGGTTCAAATG	Detection of *PSE1*	this study
FP157	ATGAAGTACACTCTCGCTACC	Detection of *FEM1*	Michielse et al. 2009[12]
FP158	GGTGAAAGTGAAAGAGTCACC	Detection of *FEM1*	Michielse et al. 2009[12]
67	TTGCAAAAGGTCGTAACAGGTT	Detection of the *SIX8-PSE1* locus	this study
41	GCGATTGTTGTATCCCTGTA	Cloning of the *SIX8-PSE1* locus and the *FomtSIX8-PSE1* locus	this study
42	TCGAAGAGTGGTGGTCAG	Cloning of the *SIX8-PSE1* locus and the *FomtSIX8-PSE1* locus	this study
373	GTTGTAGAATGTCACAGACCTAG	Cloning of the *FolSIX8-PSL1* locus	this study
374	GCTATCTATTCTAGTGGTTCG	Cloning of the *FolSIX8-PSL1* locus	this study
91	TACCCATCTCTTGGGTGTAAG	Disruption of the *SIX8-PSE1* locus	this study
92	CCCGATAGGGATAAGGAG	Disruption of the *SIX8-PSE1* locus	this study
93	TGCTCCTTATCCCTATCGGGGTCGACAGAAGATGATATTG	Disruption of the *SIX8-PSE1* locus (amplification of the *hph* cassete)	this study
94	TTACACCCAAGAGATGGGTACTAGAAAGAAGGATTACCTC	Disruption of the *SIX8-PSE1* locus (amplification of the *hph* cassete)	this study
114	ACTCGCCGATAGTGGAAAC	Confirmation of disruption of the *SIX8-PSE1* locus	this study
116	AGTAGAGGAAGGTCGGGATC	Confirmation of disruption of the *SIX8-PSE1* locus	this study
123	TACCCATCTCTTGGGTGTAAG	Transformation of *SIX8*	this study
124	GGTTGGTAACATGAGAAGG	Transformation of *SIX8*	this study
125	ACCTTCTCATGTTACCAACCGTCGACAGAAGATGATATTG	Transformation of *SIX8* (amplification of the *hph* cassete)	this study
126	TTACACCCAAGAGATGGGTACTAGAAAGAAGGATTACCTC	Transformation of *SIX8* (amplification of the *hph* cassete)	this study
119	AGCTGCCGTATATAATAGAG	Transformation of *PSE1*	this study
120	CCCGATAGGGATAAGGAG	Transformation of *PSE1*	this study
121	TGCTCCTTATCCCTATCGGGGTCGACAGAAGATGATATTG	Transformation of *PSE1* (amplification of the *hph* cassete)	this study
122	CTCTATTATATACGGCAGCTCTAGAAAGAAGGATTACCTC	Transformation of *PSE1* (amplification of the *hph* cassete)	this study
552	TTTCCGCGCAGTCAGCGTTC	qPCR for *TUB2*	this study
553	TAGCGACACGGCCACGGAAA	qPCR for *TUB2*	this study
323	ACGCGTTCTTTTACTCTTCC	qPCR for *SIX8*	this study
324	ATGCAGTGAGATAGCATCCT	qPCR for *SIX8*	this study
327	TGCAATGGTTTTGCTGTACT	qPCR for *PSE1*	this study
328	AAAAGGTCGTAACAGGTTCG	qPCR for *PSE1*	this study
414	CACCATGACGCATATTGATAAGA	Cloning of *SIX8* for yeast-two hybrid assays	this study
415	GAAATTGTTTATAAACTGGACAGTCGAT	Cloning of *SIX8* for yeast-two hybrid assays	this study
416	CACCATGGAAGACTGGGATC	Cloning of *PSE1* for yeast-two hybrid assays	this study
417	CTTGAAGCATCCATCCGA	Cloning of *PSE1* for yeast-two hybrid assays	this study

Supplementary Table 4. Plasmid DNAs used in this study.

Plasmids	Description	Resistance	Reference
pCSN43	vector for expression of *hph*	ampicillin, hygromycin B	Staben et al. 1989[13]
pMK412	vector for expression of *hph* and *GFP*	ampicillin, hygromycin B	Watanabe et al. 2007[14]
pMD-GEN	vector for expression of *nptII* and *GFP*	ampicillin, geneticin	Saito et al. 2021[15]
pSIX8-PSE1	vector for expression of *SIX8* and *PSE1*	spectinomycin	this study
pSIX8	vector for expression of *SIX8* and *hph*	spectinomycin, hygromycin B	this study
pPSE1	vector for expression of *PSE1* and *hph*	spectinomycin, hygromycin B	this study
pKOSIX8-PSE1	vector for disruption of the *SIX8-PSE1* locus	spectinomycin, hygromycin B	this study
pFomtSIX8-PSE1	vector for expression of *FomtSIX8* and *FomtPSE1*	spectinomycin	this study
pFolSIX8-PSL1	vector for expression of *FolSIX8* and *PSL1*	spectinomycin	this study
pSIX8-AD	vector for expression of *SIX8* fused with an activating domatin of *GAL4*	carbendazim	this study
pPSE1-AD	vector for expression of *PSE1* fused with an activating domatin of *GAL4*	carbendazim	this study
pSIX8-DB	vector for expression of *SIX8* fused with a DNA binding domatin of *GAL4*	carbendazim	this study
pPSE1-DB	vector for expression of *PSE1* fused with a DNA binding domatin of *GAL4*	carbendazim	this study
pDEST-AD	vector for expression of an activating domatin of *GAL4*	carbendazim	Ahmed et al. 2018[16]
pDEST-DB	vector for expression of a DNA binding domatin of *GAL4*	carbendazim	Ahmed et al. 2018[16]

Supplementary Table 5. Genome sequences used for repeat element prediction.

Organism	Isolate	N50 contig length (Mb)	GenBank assembly accession	Reference
F. oxysporum f. sp. *cubense*	160527	4.9	GCA_005930515.1	Asai et al. 2019[5]
F. oxysporum f. sp. *conglutinans*	Cong:1-1	4.9	RSAI00000000	This study
F. oxysporum f. sp. *cubense*	TR4	4.5	GCA_007994515.1	Warmington et al. 2019[4]
F. oxysporum f. sp. *radicis-cucumerinum*	*Forc* 016	4.5	GCA_001702695.2	van Dam et al. 2017[6]
F. oxysporum f. sp. *lycopersici*	race 3	4.4	GCA_003977725.1	Henry et al. 2019[17]
F. oxysporum f. sp. *melonis*	26406	4.4	GCA_002318975.1	van Dam et al. 2017[6]
F. oxysporum f. sp. *cepae*	FoC_Fus2	4.1	GCA_003615085.1	Armitage et al. 2018[7]
F. oxysporum f. sp. *lycopersici*	4287	1.3	GCA_003315725.1	Ayhan et al. 2018[18]

Supplementary References

1. Fokkens, L. *et al.* A chromosome-scale genome assembly for the *Fusarium oxysporum* strain Fo5176 to establish a model *Arabidopsis*-fungal pathosystem. *G3 (Bethesda)* **10**, 3549-3555 (2020).
2. Yu, H., Ayhan, D.H., Diener, A.C. & Ma, L.J. Genome sequence of *Fusarium oxysporum* f. sp. *matthiolae*, a Brassicaceae pathogen. *Mol. Plant. Microbe Interact.* **33**, 569-572 (2020).
3. Ma, L.J. *et al.* Comparative genomics reveals mobile pathogenicity chromosomes in *Fusarium*. *Nature* **464**, 367-373 (2010).
4. Warmington, R.J. *et al.* High-quality draft genome sequence of the causal agent of the current panama disease epidemic. *Microbiol. Resour. Announc.* **8**, e00904-19 (2019).
5. Asai, S. *et al.* High-quality draft genome sequence of *Fusarium oxysporum* f. sp. *cubense* Strain 160527, a causal agent of panama disease. *Microbiol. Resour. Announc.* **8**, e00654-19 (2019).
6. van Dam, P. *et al.* A mobile pathogenicity chromosome in *Fusarium oxysporum* for infection of multiple cucurbit species. *Sci. Rep.* **7**, 9042 (2017).
7. Armitage, A.D. *et al.* Characterisation of pathogen-specific regions and novel effector candidates in *Fusarium oxysporum* f. sp. *cepae*. *Sci. Rep.* **8**, 13530 (2018).
8. Seo, S., Pokhrel, A. & Coleman, J.J. The genome sequence of five genotypes of *Fusarium oxysporum* f. sp. *vasinfectum*: a resource for studies on Fusarium wilt of cotton. *Mol. Plant. Microbe Interact.* **33**, 138-140 (2020).
9. Kawabe, M. *et al.* Three evolutionary lineages of tomato wilt pathogen, *Fusarium oxysporum* f. sp. lycopersici, based on sequences of *IGS*, *MAT1*, and *pg1*, are each composed of isolates of a single mating type and a single or closely related vegetative compatibility group. *J. Gen. Plant Pathol.* **71**, 263-272 (2005).
10. Kashiwa, T. *et al.* An avirulence gene homologue in the tomato wilt fungus *Fusarium oxysporum* f. sp *lycopersici* race 1 functions as a virulence gene in the cabbage yellows fungus *F. oxysporum* f. sp *conglutinans*. *J. Gen. Plant Pathol.* **79**, 412-421 (2013).
11. Zhang, P., Legris, G., Coulin, P. & Puonti-Kaerlas, J. Production of stably transformed cassava plants via particle bombardment. *Plant Cell Rep.* **19**, 939-945 (2000).
12. Michielse, C.B. *et al.* The nuclear protein Sge1 of *Fusarium oxysporum* is required for parasitic growth. *PLoS Pathog.* **5**, e1000637 (2009).
13. Staben, C. *et al.* Use of a bacterial hygromycin B resistance gene as a dominant selectable marker in *Neurospora crassa* transformation. *Fungal Genet. Rep.* **36**, Article 22 (1989).
14. Watanabe, S. *et al.* Mode of action of *Trichoderma asperellum* SKT-1, a biocontrol agent against *Gibberella fujikuroi*. *J. Pestic. Sci.* **32**, 222-228 (2007).
15. Saito, H. *et al.* Spray application of nonpathogenic fusaria onto rice flowers controls bakanae disease (caused by *Fusarium fujikuroi*) in the next plant generation. *Appl. Environ. Microbiol.* **87**,

e01959-20 (2021).

16. Ahmed, H. *et al.* Network biology discovers pathogen contact points in host protein-protein interactomes. *Nat. Commun.* **9**, 2312 (2018).

17. Henry, P.M. *et al.* Genome sequence of a California isolate of *Fusarium oxysporum* f. sp. *lycopersici* race 3, a fungus causing wilt disease on tomato. *Microbiol. Resour. Announc.* **8**, e01713-18 (2019).

18. Ayhan, D.H., Lopez-Diaz, C., Di Pietro, A. & Ma, L.J. Improved assembly of reference genome *Fusarium oxysporum* f. sp. *lycopersici* strain Fol4287. *Microbiol. Resour. Announc.* **7**, e00910-18 (2018).
