# Supplementary material for: A pair of effectors encoded on a conditionally dispensable chromosome of Fusarium oxysporum suppress host-specific immunity
Source: Commun Biol. 2021 Jun 9;4:707. doi: 10.1038/s42003-021-02245-4 (PMC8190069; doi:10.1038/s42003-021-02245-4)
Supplement: Supplementary file 3 — Description of additional supplementary files [file 42003_2021_2245_MOESM3_ESM.pdf]

Description of Additional Supplementary Files

File name: Supplementary Data 1

Description: List and expression levels of FocnCong:1-1 high confidence effector candidates.

File name: Supplementary Data 2

Description: Expression levels of FocnCong:1-1 genes.

File name: Supplementary Data 3

Description: The source data underlying Figures 2c, d, 3b, c, 4a, b, d, 5a, c, d and 6c.
